# PINK1-Dependent Mitophagy Inhibits Elevated Ubiquitin Phosphorylation Caused by Mitochondrial Damage

**DOI:** 10.1021/acs.jmedchem.3c00555

**Published:** 2023-05-30

**Authors:** Olivia A. Lambourne, Shane Bell, Léa P. Wilhelm, Erika B. Yarbrough, Gabriel G. Holly, Oliver M. Russell, Arwa M. Alghamdi, Azeza M. Fdel, Carmine Varricchio, Emma L. Lane, Ian G. Ganley, Arwyn T. Jones, Matthew S. Goldberg, Youcef Mehellou

**Affiliations:** † Cardiff School of Pharmacy and Pharmaceutical Sciences, Cardiff University, Cardiff CF10 3NB, U.K.; ‡ Wellcome Centre for Mitochondrial Research, Newcastle University, Tyne NE2 4HH, U.K.; § MRC Protein Phosphorylation and Ubiquitylation Unit, University of Dundee, Dundee DD1 4HN, U.K.; ∥ Center for Neurodegeneration and Experimental Therapeutics, Department of Neurology, The University of Alabama at Birmingham, Birmingham, Alabama 35294, United States

## Abstract

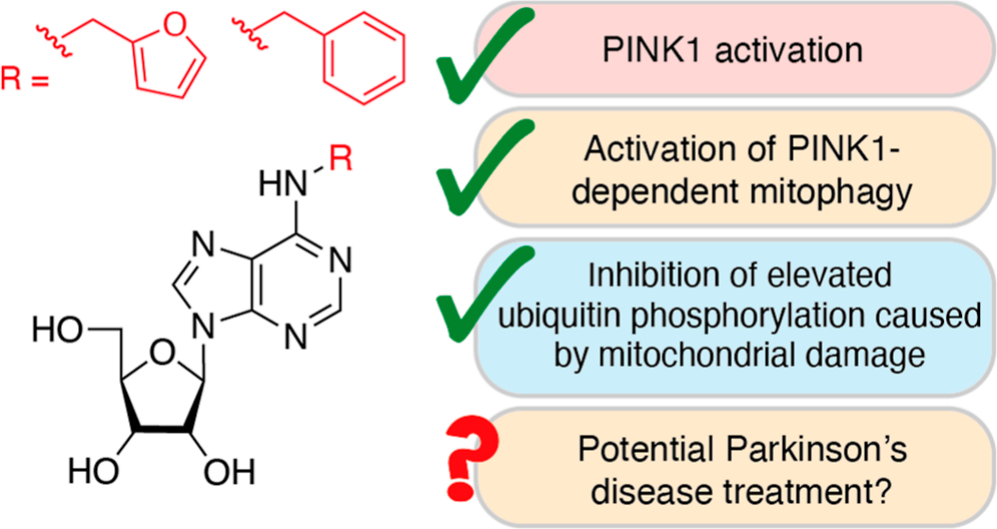

Ubiquitin phosphorylation by the mitochondrial protein kinase PTEN-induced kinase 1 (PINK1), upon mitochondrial depolarization, is an important intermediate step in the recycling of damaged mitochondria via mitophagy. As mutations in PINK1 can cause early-onset Parkinson’s disease (PD), there has been a growing interest in small-molecule activators of PINK1-mediated mitophagy as potential PD treatments. Herein, we show that *N*^6^-substituted adenosines, such as *N*^6^-(2-furanylmethyl)adenosine (known as kinetin riboside) and *N*^6^-benzyladenosine, activate PINK1 in HeLa cells and induce PINK1-dependent mitophagy in primary mouse fibroblasts. Interestingly, pre-treatment of HeLa cells and astrocytes with these compounds inhibited elevated ubiquitin phosphorylation that is induced by established mitochondrial depolarizing agents, carbonyl cyanide *m*-chlorophenyl-hydrazine and niclosamide. Together, this highlights *N*^6^-substituted adenosines as progenitor PINK1 activators that could potentially be developed, in the future, as treatments for aged and sporadic PD patients who have elevated phosphorylated ubiquitin levels in the brain.

## Introduction

**Figure 1:**
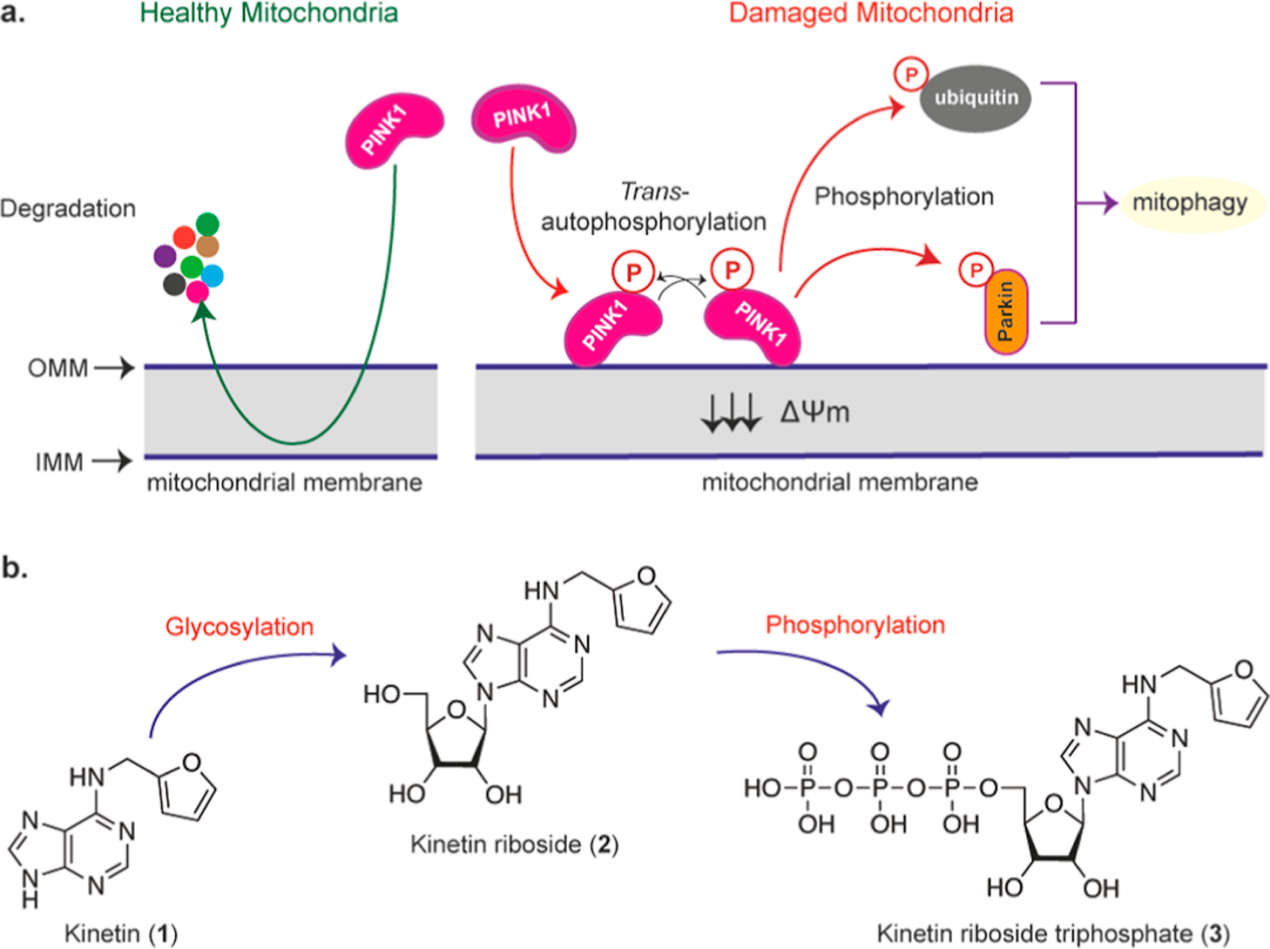
PINK1 signaling and small-molecule modulators. (a) Schematic representation of PINK1/Parkin signaling in healthy and damaged mitochondria. Figure reproduced from Lambourne and Mehellou^15^ with some modifications. (b) Chemical structure of kinetin and its metabolism to generate the PINK1 ATP-neosubstrate, KR triphosphate.

Mitochondrial serine/threonine PTEN-induced kinase 1 (PINK1) has emerged as a key player in mitochondrial quality control.^1^ In healthy mitochondria, PINK1 is constitutively recruited to the outer mitochondrial membrane where it undergoes N-terminal cleavage by proteases^2^ and subsequent proteasomal degradation in the cytosol (Figure 1a).^3^ However, in damaged mitochondria and following depolarization of the inner mitochondrial membrane, PINK1 gets stabilized on the outer mitochondrial membrane in its full-length form.^4^ Accumulation of PINK1 results in trans-autophosphorylation and its subsequent activation.^5^ Active PINK1 then phosphorylates the E3 ubiquitin ligase Parkin at serine 65^6^ and also ubiquitin at serine 65.^7^ This ultimately results in the ubiquitylation of various proteins on the outer mitochondrial membrane, leading to mitochondrial degradation by the autophagic machinery.^8^

PINK1 kinase activity has been highlighted as being vital in preventing the development of neurodegeneration, exemplified by its loss-of-function mutations, causing a form of early-onset Parkinson’s disease (PD).^9^ This observation led to the discovery that kinetin, an *N*^6^-substituted adenine (**1**, Figure 1b), enhanced PINK1 activation in cells when exposed to the mitochondrial de-polarizing agent carbonyl cyanide *m*-chlorophenyl-hydrazine^10^ (CCCP).^11^ Kinetin activation of PINK1 was noted to be due to its bioconversion to the active metabolite kinetin riboside (KR) triphosphate (**3**, Figure 1b), which acts as an ATP-neosubstrate for PINK1.^11^ With this observation in mind and as a result of our interest in developing nucleoside analogue therapeutics,^12^ we subsequently showed that the nucleoside derivative of kinetin, called KR (**2**, Figure 1b), exhibited more potent CCCP-independent activation of PINK1 in cells compared to its nucleobase derivative, kinetin, as evidenced by Parkin serine 65 phosphorylation.^13^ Herein, we report on the effect of KR and other nucleoside analogues on niclosamide-^14^ and CCCP-induced ubiquitin phosphorylation and PINK1-dependent mitophagy.

## Results and Discussion

**Figure 2:**
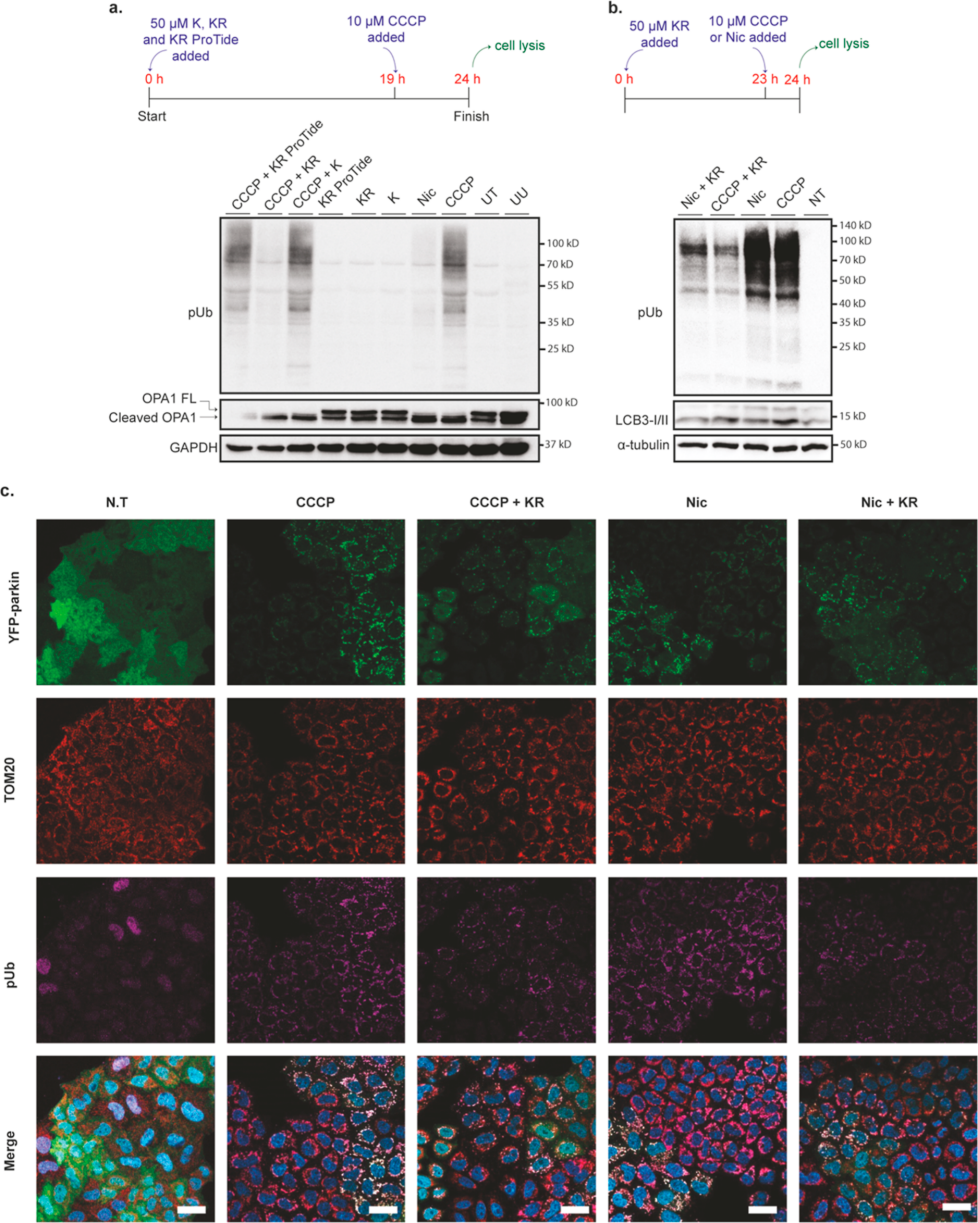
KR suppresses niclosamide- and CCCP-induced ubiquitin serine 65 phosphorylation. (a) HeLa cells transfected with Parkin were pre-treated with 50 μM KR for 24 h, and then, they were either lysed or treated with 10 μM CCCP for 3 h. The cell lysates were probed for pUb, OPA1, and GAPDH. UU: untreated and untransfected; UT: untreated and transfected. (b) HeLa cells transfected with YFP-Parkin were pre-treated with 50 μM KR for 24 h, and then, they were either lysed or treated with 10 μM niclosamide or CCCP for 1 h. The cell lysates were probed for pUb, LC3B-I/-II, and α-tubulin. N.T.: non-treated cells. (c) As in (b) but samples studied using immunofluorescence for pUb, YFP-Parkin expression, and TOM20. Scale bar = 40 μm.

The reported PINK1 activation by KR in cells was noted at a high concentration (50 μM),^13^ and detectable PINK1 activation in cells by the nucleobase kinetin was only observed in the presence of the mitochondrial uncoupler CCCP.^11^ Therefore, we initially asked whether combining this nucleoside analogue, KR, with some of the known indirect^15^ PINK1 activators such as niclosamide and CCCP would lead to a synergistic and more significant activation of PINK1. To explore this, we first treated Parkin-transfected HeLa cells for 24 h with 50 μM kinetin, KR, or KR ProTide,^13^ a monophosphate prodrug of KR. Subsequently, the cells were either lysed or treated with 10 μM CCCP for 3 h. Niclosamide and CCCP treatments alone were used as controls. Upon cell lysis and probing for ubiquitin serine 65 phosphorylation, optic atrophy protein 1 (OPA1) and GAPDH, niclosamide and CCCP treatments alone, as expected, produced strong phosphorylation of ubiquitin at serine 65 as judged by the formation of phosphorylated ubiquitin, pUb (Figure 2a). The treatment of cells with 50 μM kinetin, KR, or KR ProTide alone did not lead to any notable phosphorylation of ubiquitin since no phosphorylated ubiquitin bands were detected. Strikingly, pre-treatment of cells with KR followed by CCCP prevented ubiquitin phosphorylation, which was observed under the CCCP treatment alone. This, however, was not observed in the samples pre-treated with the related nucleobase kinetin or KR ProTide pre-treatment. Beyond ubiquitin phosphorylation, the treatment of cells with niclosamide and CCCP alone led to OPA1 cleavage, indicating mitochondrial membrane depolarization,^16^ while pre-treatment with KR did not prevent this effect (Figure 2a). Next, we explored if this same effect KR had on the CCCP-induced ubiquitin phosphorylation could also be observed with the mitochondrial uncoupler niclosamide. For this, HeLa cells transfected with YFP-Parkin were pre-treated with 50 μM KR for 24 h and then were either lysed or treated with 10 μM niclosamide or CCCP for 1 h. Again, and as expected, the control samples in which the cells were treated with only niclosamide and CCCP induced strong ubiquitin phosphorylation, as judged by the formation of phosphorylated ubiquitin, whereas the pre-treatment of cells with KR inhibited both the niclosamide- and CCCP-induced phosphorylation of ubiquitin (Figure 2b) similar to the outcome in Figure 2a.

The ability of KR to inhibit CCCP- and niclosamide-mediated ubiquitin phosphorylation was then probed using immunofluorescence (Figure 2c). YFP-Parkin-transfected HeLa cells were either left untreated or treated with 10 μM niclosamide or 10 μM CCCP alone for 1 h or pre-treated with 50 μM KR for 24 h prior to the addition of niclosamide or CCCP, as illustrated in Figure 2b. Treatment of cells with niclosamide and CCCP again induced significant phosphorylation of ubiquitin, while pre-treatment of cells with KR inhibited ubiquitin phosphorylation (Figure 2c). Although pre-treatment with KR inhibited ubiquitin phosphorylation, it did not prevent the membrane potential collapse caused by niclosamide and CCCP (Supporting Information, Figure S1) akin to the observation noted by probing for OPA1 cleavage (Figure 2a).

**Scheme 1:**
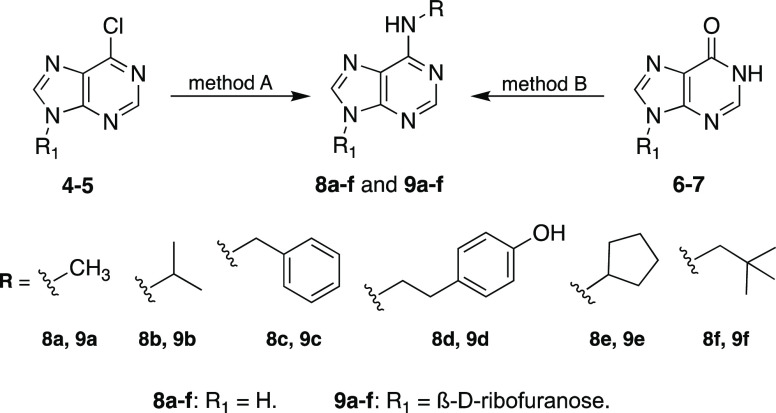
Chemical Synthesis of *N*^6^-Substituted Adenines and Adenosines and Their Activation of PINK1 in Cells Reagents and conditions. Method A: triethylamine, ethanol, heating, 16 h. Method B: PyBOP, DIPEA, acetonitrile/DMF, 3 days, rt.

In view of the ability of KR to inhibit niclosamide- and CCCP-induced ubiquitin phosphorylation, we subsequently designed and synthesized *N*^6^-substituted adenines and adenosines that are structurally related to kinetin and KR. In the design of these nucleobases and nucleosides, we elected to modify the *N*^6^-position of adenine and adenosine and chose to make various small, medium, and bulky substitutions at this position. Precisely, the *N*^6^-substitutions were methyl, isopropyl, benzyl, tyramine, cyclopentylamine, neopentylamine, and furfuryl (**8a–f** and **9a–f**, Scheme 1).

The synthesis of the *N*^6^-substituted adenine and adenosine compounds was carried out using one of the two methods depending on the volatility of the nucleophile being used in the reaction (Scheme 1). Standard nucleophilic S_N2_ substitution was the preferred method (method A) as it gave high yields after a simple purification step. This method involved heating 6-chloropurine or its nucleoriboside derivative with the corresponding nucleophile in ethanol in the presence of triethylamine. This method was employed in the synthesis of the adenine and adenosine analogues that had methyl (**8a** and **9a**), isopropyl (**8b** and **9b**), benzyl (**8c** and **9c**), or tyramine (**8d**) modifications at the *N*^6^-position. In method B, the peptide coupling agent benzotriazol-1-yloxytripyrrolidinophosphonium hexafluorophosphate (PyBOP) was used for the synthesis of nucleobases and nucleosides bearing tyramine (**9d**), cyclopentylamine (**8e** and **9e**), and neopentylamine (**8f** and **9f**) at the *N*^6^-position. The choice to employ method B for the synthesis of these compounds was driven by the fact that the nucleophiles used are explosive when heated, ruling out using method A. For the synthesis of the nucleobases and nucleosides in this case, hypoxanthine or inosine was added to PyBOP and partially dissolved in a mixture of acetonitrile and substoichiometric quantities of DMF. The corresponding nucleophiles, cyclopentylamine or neopentylamine, were then added and left to react for 3 days. Compared to method A, method B gave much lower yields, and the purification of the final compounds was more complicated.

**Figure 3:**
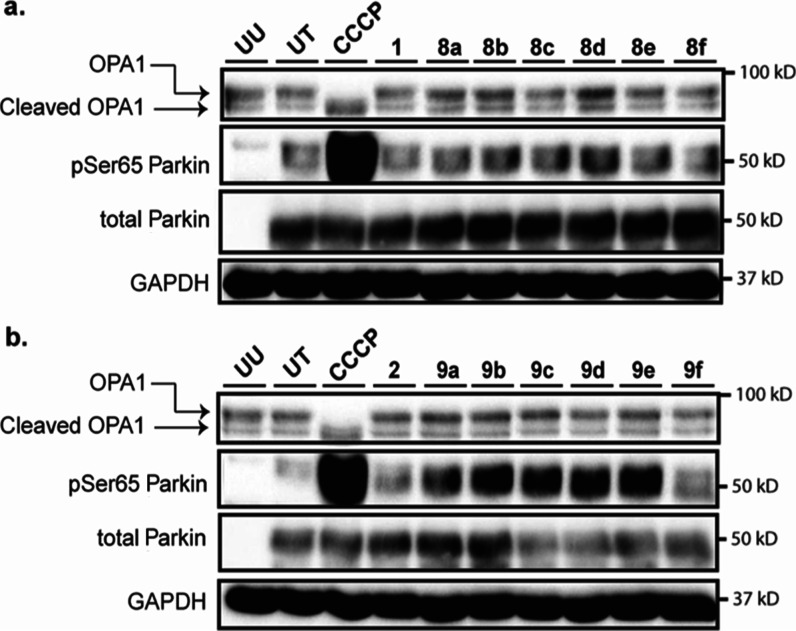
Activation of PINK1 by *N*^6^-substituted adenines and adenosines. HeLa cells transfected with Parkin were treated with 50 μM **1**, **2**, **8a–8f** (a), and **9a–9f** (b) for 1 h. CCCP was used at 10 μM, and the treatment was for 3 h. Cells were then lysed and probed for Parkin serine 65 phosphorylation (pSer65 Parkin), total Parkin, OPA1, and GAPDH. UU: untreated and untransfected HeLa cells. UT: untreated and Parkin-transfected HeLa cells. The data are representative of three repeat experiments.

Upon the synthesis of these nucleobases and nucleosides, we subsequently investigated their ability to activate PINK1. HeLa cells, which endogenously express PINK1, but not Parkin, were transiently transfected with Parkin. Subsequently, the cells underwent treatment with nucleobases (**8a–8f**) and nucleosides (**9a–9f**) for 1 h or 10 μM CCCP for 3 h as a control and probed for Parkin serine 65 phosphorylation, total Parkin, OPA1, and GAPDH as a loading control. The results showed that CCCP resulted in prominent activation of PINK1 as judged by Parkin serine 65 phosphorylation, while nucleobases **8a–8f** did not show any significant activation of PINK1 at 50 μM in agreement with our previous finding^13^ (Figure 3a). However, all of the nucleosides studied (**9a–9f**) exhibited pronounced activation of PINK1 apart from compound **9f** (Figure 3b). Interestingly, the activation of PINK1 by these nucleosides did not result in the cleavage of OPA1. This indicates that these nucleoside analogues activate PINK1 independent of mitochondrial depolarization in contrast to CCCP activation of PINK1, which was associated with cleavage of OPA1. In line with previous observations,^10^ this is because this agent, CCCP, activates PINK1 indirectly via the depolarization of the mitochondrial membrane.^10^

**Figure 4:**
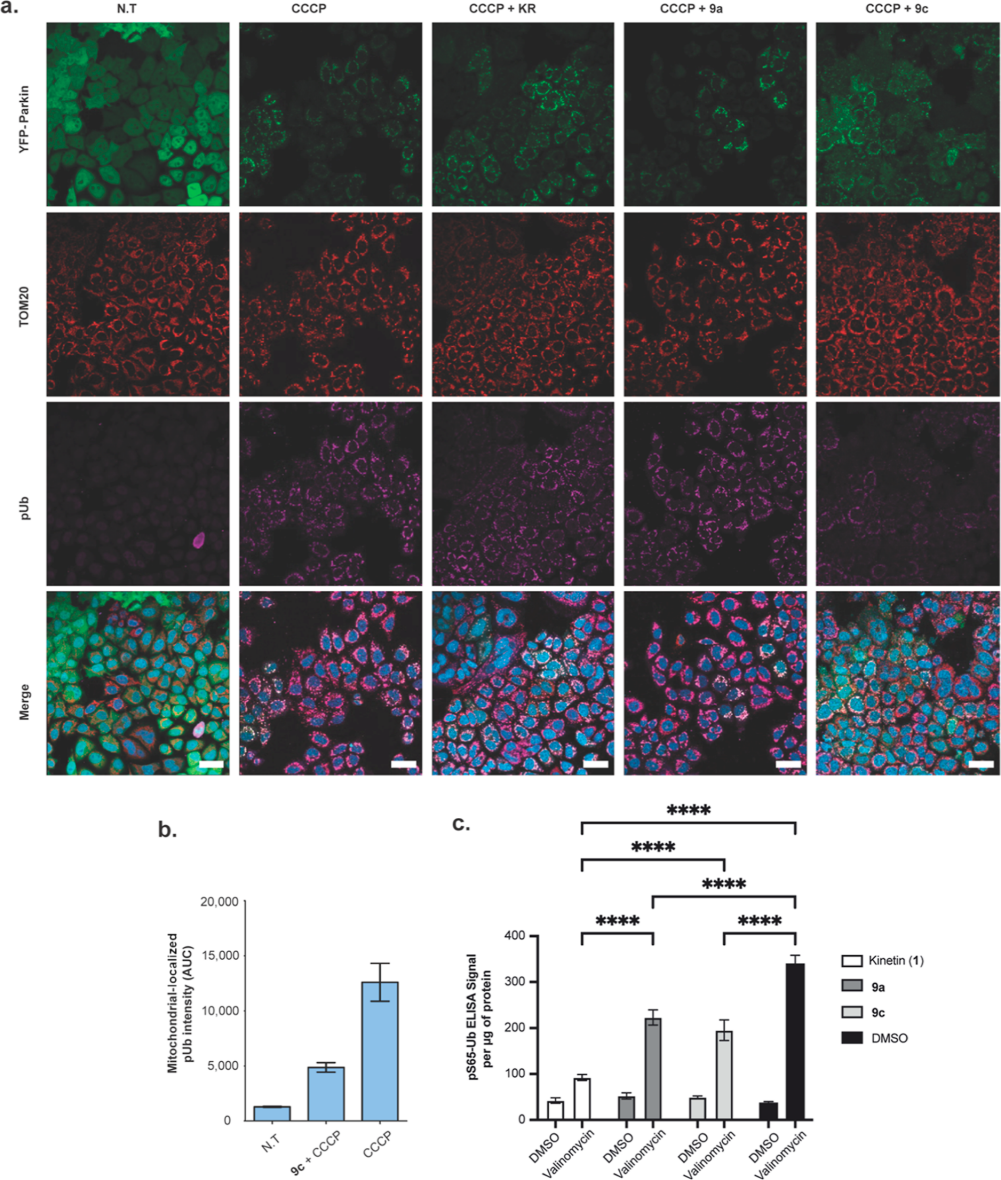
*N*^6^-benzyladenosine inhibits CCCP-mediated ubiquitin serine 65 phosphorylation in HeLa cells and astrocytes. (a) HeLa cells transfected with YFP-Parkin were pre-treated with 50 μM KR or *N*^6^-benzyladenosine for 24 h, and then, they were either lysed or treated with 10 μM CCCP for 1 h. Immunofluorescence data probing for pUb, YFP-Parkin expression, and TOM20 of the samples. Scale bar = 40 μm. (b) Quantification of ubiquitin phosphoserine 65 localization to the mitochondria. (c) ELISA quantification of ubiquitin phosphoserine 65 (pS65-Ub) in lysates of astrocytes treated with 50 μM kinetin (1), *N*^6^-methyladenosine (**9a**), and *N*^6^-benzyladenosine (**9c**) or DMSO for 24 h with 10 nM valinomycin or DMSO for the final 5 h. Data are shown as mean ± SEM ELISA signal per microgram of protein, measured by BCA assay (*n* = 8).

Next, we explored the impact of KR and nucleosides **9a** and **9c** on mitochondria in the absence of niclosamide and CCCP. First, HeLa cells transfected with YFP-Parkin were either left untreated or treated with 50 μM KR and nucleosides **9a** and **9c** for 24 h. Probing for TOM20 by immunofluorescence in these cells indicated that these nucleoside analogues alone did not have any impact on mitochondrial fragmentation, Parkin localization, or ubiquitin phosphorylation (Supporting Information, Figure S2). Subsequently, YFP-Parkin-expressing HeLa cells were treated with 50 μM KR and nucleosides **9a** or **9c** for 24 h, and this was followed by 10 μM CCCP treatment. Using immunofluorescence to monitor ubiquitin serine 65 phosphorylation, CCCP treatment induced strong ubiquitin phosphorylation and promoted Parkin localization to the mitochondria (Figure 4a,b). Notably, ubiquitin phosphorylation was inhibited by KR pre-treatment in agreement with the data (Figure 2a–c). In terms of the new compounds, pre-treatment with nucleoside analogue **9a** followed by CCCP treatment did not have a significant impact on ubiquitin phosphorylation and Parkin localization to the mitochondria compared to the CCCP treatment alone (Figure 4a). However, pre-treatment with nucleoside analogue **9c** produced significant reduction in ubiquitin phosphorylation and Parkin localization to the mitochondria (Figure 4b), which appears stronger than that induced by KR (Figure 4a).

With the consistent observation that KR and nucleoside **9c** inhibit ubiquitin phosphorylation in HeLa cells, we explored whether this effect was evident in astrocytes, the brain cell type in which PINK1 activity is most prominently observed.^17^ Briefly, primary mouse astrocytes were treated with 50 μM nucleobase kinetin (**1**), nucleosides **9a** and **9c**, or DMSO for 24 h, and 5 h prior to lysis, the samples were treated with 10 nM valinomycin, a mitochondrial uncoupler, or DMSO as a control. Quantification of ubiquitin serine 65 phosphorylation by ELISA indicated that treatment with the nucleobase kinetin (**1**) and nucleosides **9a** and **9c** alone resulted in increased phosphorylation of ubiquitin serine 65 (Figure 4c), in line with the results observed with Parkin serine 65 phosphorylation shown in Figure 3b. Interestingly, the pre-treatment of astrocytes with kinetin (**1**) and nucleosides **9a** and **9c** for 24 h followed by treatment with valinomycin for 5 h led to significant suppression of ubiquitin serine 65 phosphorylation as compared the treatment of astrocytes with valinomycin alone (black bar, Figure 4c).

**Figure 5:**
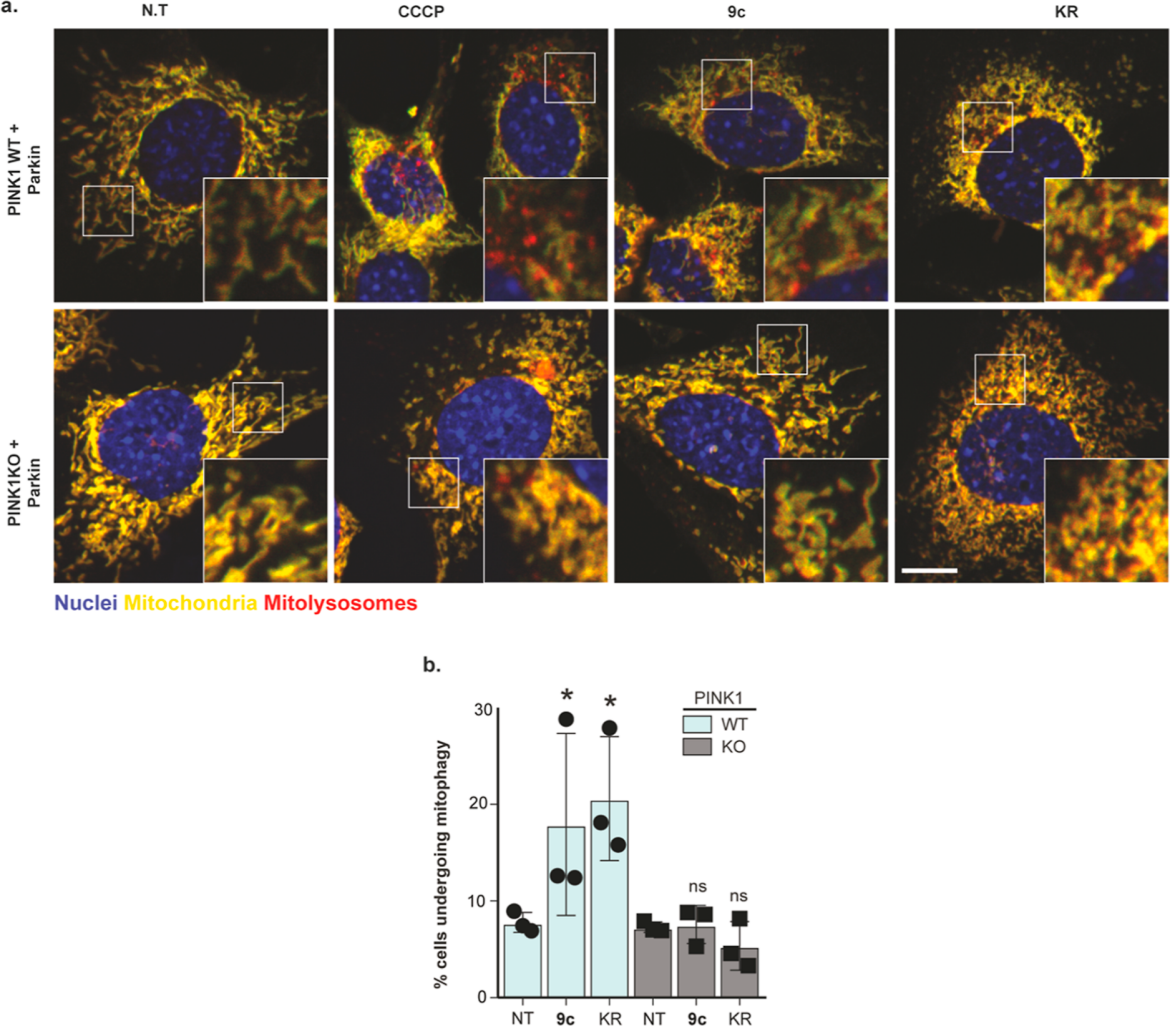
*N*^6^-benzyladenosine induces mitophagy in a PINK1-dependent manner. (a) Representative images of mitophagy in WT and PINK1 KO *mito*-QC MEFs overexpressing Parkin. Cells were treated with DMSO (N.T), 5 μM CCCP, *N*^6^-benzyladenosine (**9c**), and KR for 16 h. *n* = 3. (b) Quantification by FACS of MEFs undergoing mitophagy from (a).

With the inhibition of ubiquitin phosphorylation and Parkin localization to the mitochondria by KR and the nucleoside analogue **9c**, we next asked whether these compounds still induce mitophagy in a PINK1-dependent manner. To explore this, we employed the established *mito*-QC assay for measuring mitophagy in cells.^18^ Immortalized mouse embryonic fibroblasts (MEFs) overexpressing HA-tagged Parkin, derived from littermate PINK1 wild-type (WT) or knock-out (KO) *mito*-QC mice embryos,^19^ were treated with 20 μM CCCP or 5 μM KR or compound **9c** for 16 h. CCCP treatment induced an increase in mitophagy in WT MEFs but not in PINK1 KO MEFs, in line with previous reports^19^ (Figure 5). Interestingly, treatment of WT MEFs with KR or the nucleoside analogue **9c** also induced mitophagy, while this was not observed in PINK1 KO MEFs (Figure 5). These data indicate that both KR and the nucleoside analogue **9c** induce a low level of mitophagy in a PINK1-dependent manner, which is in line with the previous observation that KR^13^ and nucleoside **9c** induce low-level activation of PINK1 as judged by Parkin serine 65 phosphorylation (Figure 3).

Together, these results showcase the ability of KR and *N*^6^-benzyladenosine (**9c**) to inhibit CCCP- and niclosamide-induced ubiquitin phosphorylation. To the best of our knowledge, there have been two examples of inhibiting PINK1’s ability to phosphorylate ubiquitin.^20,21^ The first concerns PD-associated mutations C125G and Q126P that disrupt an intramolecular interaction between the human PINK1’s N- and C-terminal extensions, leading to the inhibition of ubiquitin phosphorylation,^21^ while the second is the oxidation of human PINK1 Cys166 and/or Cys387.^20^ Inspection of the AlphaFold human PINK1 structure and molecular modeling studies (Supporting Information, Figure S3) indicated that these cysteine residues are remote from the binding site of the nucleobase of the nucleoside analogues and their triphosphate derivatives, and it is, therefore, unlikely that these can be covalent inhibitors. To examine this further, we ran in vitro kinase assays employing recombinant and constitutively active *Tribolium castaneum* PINK1 (*Tc*PINK1)^22^ and either human Parkin or ubiquitin as substrates in the presence of increasing concentrations (max 100 μM) of KR, benzyladenosine **9c**, or its triphosphate derivative. The results indicated that these compounds showed no inhibition of *Tc*PINK1’s ability to phosphorylate Parkin or ubiquitin in vitro (Supporting Information, Figure S4) and hence are unlikely to be covalent inhibitors. Similar data were obtained using in vitro kinase assay employing *Tc*PINK1 and recombinant human ubiquitin (Supporting Information, Figure S5). It must be noted though that the human PINK1 Cys166 is not conserved in *Tc*PINK1, while Cys387 is conserved across human PINK1 and *Tc*PINK1 (Supporting Information, Figure S6). Additionally, the superimposition of the ATP pockets of the hPINK1 and *Tc*PINK1 showed the *Tc*PINK1 ATP pocket to be more open than that of hPINK1. Furthermore, although the position of hPINK1 Cys387 was close to that of the *Tc*PINK1 Cys362, the position of the *Tc*PINK1 Thr172 was remote from that of hPINK1 Cys66 (Supporting Information, Figure S7), which appears to have an impact on hPINK1’s ability to phosphorylate ubiquitin. In terms of the second possibility of KR and **9c** disrupting the intramolecular interaction between PINK1’s N- and C-terminal extensions akin to the Q126P mutation,^21^ this may result from the binding of these compounds or their phosphorylated species to the ATP pocket of PINK1 causing a conformational change that rearranges the N- and C-ends, leading to the inability of PINK1 to phosphorylate ubiquitin.

In terms of small molecules being able to activate PINK1 and induce PINK1-dependent mitophagy that is accompanied by a reduction in phosphorylated ubiquitin, to the best of our knowledge, no precedence of such molecules has been reported in the literature. However, while developing and deliberating on these hypotheses, a preprint was published that offered some insights into the ability of small molecules, namely, *N*^6^-substituted adenines, to induce PINK1 activation, trigger PINK1-dependent mitophagy, and clear the accumulation of phosphorylated ubiquitin in cells and in vivo.^23^ Although these *N*^6^-substituted adenines, exemplified by MTK478 (structure not shown), were found to exhibit their pharmacological activity without being converted to their corresponding nucleosides and nucleotides, they stabilized the active form of PINK1.^23^ This in turn triggered PINK1-dependent mitophagy and led to the clearance of phosphorylated ubiquitin. It is worth noting that in this study,^23^ the authors noted that treatment of cells with mitochondrial depolarizing agents, such as CCCP, resulted in stalled mitophagy that was accompanied by the accumulation of phosphorylated ubiquitin. With this in mind, it is plausible that the pre-treatment of cells with our nucleoside analogues activate PINK1 and trigger PINK1-dependent mitophagy, which prevents the accumulation of phosphorylated ubiquitin that results from the stalled mitophagy caused by the treatment with CCCP and niclosamide alone, as shown in Figure 2. Although we, in this work, noted this as inhibition of ubiquitin phosphorylation, given the reduction of phosphorylated ubiquitin bands (Figure 2), this may in fact be a result of our compound activation of PINK1-dependent mitophagy, clearing the elevated phosphorylated ubiquitin levels caused by CCCP and niclosamide akin to the observation made by Chin et al.^23^ This, however, will be the focus of our future studies into understanding the exact mechanism of action of these compounds.

It is also worth noting that for the *N*^6^-substituted adenines, e.g., MTK478, to stabilize the active form of PINK1, a mitochondrial suppressor, e.g., carbonyl cyanide *p*-(trifluoromethoxy)phenylhydrazone (FCCP), was required.^23^ This, again, fits with data shown here as our nucleoside analogues were able to suppress the formation of high levels of phosphorylated ubiquitin when the cells were challenged with the mitochondrial suppressors CCCP, niclosamide, or valinomycin.

## Conclusions

We herein described nucleoside analogues as activators of PINK1-dependent mitophagy, which consequently inhibit ubiquitin phosphorylation that is caused by mitochondrial uncouplers, CCCP, niclosamide, and valinomycin. Although the exact mechanism of action of these nucleoside analogues is not fully elucidated, it has recently been reported that small molecules, such as *N*^6^-substituted adenines, when used with a mitochondrial stressor activate PINK1 by stabilizing the active form of PINK1, which ultimately triggers PINK1-dependent mitophagy.^23^ Since treatment of cells with mitochondrial stressors, such as CCCP, alone is now understood to lead to stalled mitophagy that is associated with the accumulation of phosphorylated ubiquitin,^23^ PINK1 activators that trigger PINK1-dependent mitophagy ensure the completion of the mitophagy cycle and the clearance of elevated phosphorylated ubiquitin levels.

Together, this work highlights the potential use of PINK1 activators that induce PINK1-dependent mitophagy and prevent high levels of phosphorylated ubiquitin caused by mitochondrial damaging agents as treatments of idiopathic PD. This is because PD-causing α-synuclein pathology results in the accumulation of phosphorylated ubiquitin due to impaired mitophagy, and this ultimately causes neuronal death.^23^ Additionally, post-mortem analysis of the substantia nigra of these patients revealed elevated phosphorylated ubiquitin levels compared with healthy age-matched controls.^24^

## Experimental Section

### Chemistry

All reagents and solvents were of general purpose or analytical grade and were purchased from Sigma-Aldrich Ltd., Fisher Scientific, Fluorochem, or Acros. ^1^H and ^13^C NMR data were recorded on a Bruker AVANCE DPX500 spectrometer operating at 202, 500, and 125 MHz. Chemical shifts (δ) are quoted in ppm, and *J* values are quoted in Hz. In reporting spectral data, the following abbreviations were used: s (singlet), d (doublet), t (triplet), q (quartet), dd (doublet of doublets), td (triplet of doublets), and m (multiplet). All of the reactions were carried out under a nitrogen atmosphere and were monitored using analytical thin-layer chromatography on precoated silica plates (Kieselgel 60 F254, BDH). Compounds were visualized by illumination under UV light (254 nm) or by the use of KMnO_4_ stain followed by heating. Flash column chromatography was performed with silica gel 60 (230–400 mesh) (Merck). High-performance liquid chromatography (HPLC) was carried out on a SHIMADZU Prominence-i quaternary low-pressure gradient pump with a Prominence-i UV detector (190 to 700 nm). All solvents for HPLC were of HPLC grade and purchased from Fisher Scientific. HPLC data analysis was performed using the SHIMADZU Lab Solutions software package. The purity of the final compounds was determined by HPLC, and they were all of ≥95% purity unless stated otherwise.

#### *N*-Methyl-9*H*-purin-6-amine (**8a**)

Methylamine (0.40 mL, 9.01 mmol, 2.8 equiv) and TEA (0.45 mL, 3.23 mmol, 1 equiv) were added to a stirring solution of 6-chloropurine (500 mg, 3.23 mmol, 1 equiv) in EtOH (15 mL). The reaction was then heated to 30 °C for 16 h. The solvent was then removed under reduced pressure. The crude oil that remained was then purified by column chromatography using DCM/MeOH (19:1) as an eluent to give a white solid (200 mg, 41%); ^1^H NMR (500 MHz, DMSO): δ 12.93 (1H, s, NH), 8.25 (1H, s), 8.13 (1H, s), 7.60 (1H, s, NH), 3.02 (3H, s); ^13^C NMR (126 MHz, DMSO): δ 152.90, 152.85, 27.40; HRMS–ES (*m*/*z*); found [M + H]^+^, 150.0781 [C_6_H_7_N_5_H] requires 150.0780. HPLC (reverse phase) 0.5 mL/min MeOH/H_2_O 80:20 in 12 min, λ = 254 nm, Rt = 4.88 min (99%).

#### *N*-Isopropyl-9*H*-purin-6-amine (**8b**)

Isopropylamine (0.28 mL, 3.29 mmol, 1 equiv) and TEA (0.45 mL, 3.23 mmol, 1 equiv) were added to a stirring solution of 6-chloropurine (500 mg, 3.23 mmol, 1 equiv) in EtOH (15 mL). The reaction was then heated to 40 °C for 16 h. The solvent was then removed under reduced pressure. The crude oil that remained was then purified by column chromatography using DCM/MeOH (19:1) as an eluent to give a white solid (11.7 mg, 2%); ^1^H NMR (500 MHz, MeOD): δ 8.21 (1H, s), 8.06 (1H, s), 3.25–3.03 (1H, m), 1.32 (6H, d, *J* = 6.5 Hz); ^13^C NMR (126 MHz, MeOD): δ 46.52, 7.82. HPLC (reverse phase) 0.5 mL/min MeOH/H_2_O 90:10 in 12 min, λ = 254 nm, Rt = 4.69 min (99%).

#### *N*-Benzyl-9*H*-purin-6-amine (**8c**)

Benzylamine (0.42 mL, 3.88 mmol, 1.2 equiv) and TEA (0.54 mL, 3.88 mmol, 1.2 equiv) were added to a stirring solution of 6-chloropurine (500 mg, 3.24 mmol, 1 equiv) in EtOH (15 mL). The reaction was then refluxed at 80 °C for 16 h. The product crashed out of solution when placed in an ice water bath and stirred vigorously. OL035 was then filtered off and further purified by column chromatography using DCM/MeOH (19:1) as an eluent to give a white solid (240 mg, 33%); ^1^H NMR (500 MHz, MeOD): δ 8.25 (1H, s), 8.07 (1H, s), 7.39 (2H, d, *J* = 7.6 Hz), 7.32 (2H, t, *J* = 7.5 Hz), 7.25 (1H, t, *J* = 7.3 Hz); ^13^C NMR (126 MHz, MeOD): δ 152.42, 128.18, 127.18, 126.88, 46.48; HRMS–ES (*m*/*z*); found [M + H]^+^, 226.1089 [C_12_H_11_N_5_H] requires 226.1093. HPLC (reverse phase) 0.5 mL/min MeOH/H_2_O 80:20 in 12 min, λ = 254 nm, Rt = 5.63 min (99%).

#### 4-(2-((9*H*-Purin-6-yl)amino)ethyl)phenol (**8d**)

Tyramine (533 mg, 3.88 mmol, 1.2 equiv) and TEA (0.54 mL, 3.88 mmol, 1.2 equiv) were added to a stirring solution of 6-chloropurine (500 mg, 3.24 mmol, 1 equiv) in EtOH (15 mL). The reaction was then refluxed at 80 °C for 16 h. The product crashed out of solution when placed in an ice water bath and stirred vigorously. OL037 was then filtered off and further purified by column chromatography using DCM/MeOH (19:1) as an eluent to give a white solid (141 mg, 17%); ^1^H NMR (500 MHz, DMSO): δ 8.20 (1H, s), 8.07 (1H, s), 7.05 (2H, d, *J* = 7.8 Hz), 6.69 (2H, d, *J* = 8.5 Hz), 3.64–3.63 (2H, m) 2.81–2.78 (2H, m); ^13^C NMR (126 MHz, DMSO): δ 156.09, 130.04, 129.96, 115.58, 45.80, 36.53; HRMS–ES (*m*/*z*); found [M + H]^+^, 256.1205 [C_13_H_13_N_5_OH] requires 256.1198. HPLC (reverse phase) 0.5 mL/min MeOH/H_2_O 80:20 in 12 min, λ = 254 nm, Rt = 4.96 min (86%).

#### *N*-Cyclopentyl-9*H*-purin-6-amine (**8e**)

Hypoxanthine (500 mg, 3.67 mmol, 1 equiv), cyclopentylamine (0.54 mL, 5.51 mmol, 1.5 equiv), and PyBOP (2.294 g, 4.41 mmol, 1.2 equiv) were dissolved in anhydrous ACN (20 mL) and substoichiometric amounts of DMF (2 mL) under an inert atmosphere. DIPEA (1.28 mL, 7.35 mmol, 2 equiv) was then added slowly over 5 min at RT, and the reaction was left to stir for 3 days. The solvent was then removed under reduced pressure. The crude oil that remained was then purified by column chromatography using DCM/MeOH (19:1) as an eluent to give a white solid (130 mg, 17%); ^1^H NMR (500 MHz, MeOD): δ 8.23 (1H, s), 8.07 (1H, s), 2.14–2.07 (1H, m), 1.85–1.60 (8H, m); ^13^C NMR (126 MHz, DMSO): δ 152.80, 32.77, 23.91. HRMS–ES (*m*/*z*); found [M + H]^+^, 204.1244 [C_10_H_13_N_5_H] requires 204.1249. HPLC (reverse phase) 0.5 mL/min MeOH/H_2_O 80:20 in 12 min, λ = 254 nm, Rt = 5.78 min (99%).

#### *N*-Neopentyl-9*H*-purin-6-amine (**8f**)

Hypoxanthine (300 mg, 2.20 mmol, 1 equiv), neopentylamine (0.39 mL, 3.31 mmol, 1.5 equiv), and PyBOP (1.721 g, 3.31 mmol, 1.5 equiv) were dissolved in anhydrous ACN (20 mL) and substoichiometric amounts of DMF (2 mL) under an inert atmosphere. DIPEA (0.77 mL, 4.41 mmol, 2 equiv) was then added slowly over 5 min at RT, and the reaction was left to stir for 3 days. The solvent was then removed under reduced pressure. The crude oil that remained was then purified by column chromatography using DCM/MeOH (19:1) as an eluent to give a white solid (31 mg, 7%); ^1^H NMR (500 MHz, MeOD): δ 8.74 (1H, s), 8.58 (1H, s), 3.19–3.15 (2H, m), 1.89–1.84 (9H, m), ^13^C NMR (126 MHz, MeOD): δ 176.56, 151.66, 151.61, 145.74, 70.13, 25.95. HPLC (reverse phase) 0.5 mL/min MeOH/H_2_O 90:10 in 12 min, λ = 254 nm, Rt = 4.72 min (99%).

#### (2*R*,3*S*,4*R*,5*R*)-2-(Hydroxymethyl)-5-(6-(methylamino)-9*H*-purin-9-yl)tetrahydrofuran-3,4-diol (**9a**)

Methylamine (0.26 mL, 5.86 mmol, 2.8 equiv) and TEA (0.29 mL, 2.08 mmol, 1 equiv) were added to a stirring solution of 6-chloropurine riboside (600 mg, 2.09 mmol, 1 equiv) in EtOH (15 mL). The reaction was then heated to 30 °C for 16 h. The solvent was then removed under reduced pressure. The crude oil that remained was then purified by column chromatography using DCM/MeOH (19:1) as an eluent to give a white solid (274 mg, 73%); ^1^H NMR (500 MHz, MeOD): δ 8.23 (2H, s), 5.95 (1H, d, *J* = 6.5 Hz), 4.74 (1H, dd, *J* = 6.4, 5.1 Hz), 4.32–4.31 (1H, m), 4.16 (1H, q, *J* = 2.5 Hz), 3.90–3.72 (2H, m), 1.28 (3H, m); ^13^C NMR (126 MHz, MeOD): δ 89.89, 86.83, 74.05, 71.32, 62.13, 7.89; LCMS–ES (*m*/*z*); found [M + H]^+^, 282.10 [C_11_H_15_N_5_O_4_H] requires 282.11. HPLC (reverse phase) 0.5 mL/min MeOH/H_2_O 90:10 in 12 min, λ = 254 nm, Rt = 4.73 min (99%).

#### (2*R*,3*S*,4*R*,5*R*)-2-(Hydroxymethyl)-5-(6-(isopropylamino)-9*H*-purin-9-yl)tetrahydrofuran-3,4-diol (**9b**)

Isopropylamine (0.18 mL, 2.11 mmol, 1 equiv) and TEA (0.29 mL, 2.08 mmol, 1 equiv) were added to a stirring solution of 6-chloropurine riboside (600 mg, 2.09 mmol, 1 equiv) in EtOH (15 mL). The reaction was then heated to 40 °C for 16 h. The solvent was then removed under reduced pressure. The crude oil that remained was then purified by column chromatography using DCM/MeOH (19:1) as an eluent to give a white solid (471 mg, 73%); ^1^H NMR (500 MHz, MeOD): δ 8.24 (1H, s), 8.21 (1H, s), 5.94 (1H, d, *J* = 6.5 Hz), 4.74 (1H, dd, *J* = 6.4, 5.1 Hz), 4.31 (1H, m), 4.16 (1H, m), 3.81 (2H, m), 1.31 (6H, d, *J* = 6.5 Hz); ^13^C NMR (126 MHz, MeOD): δ 152.17, 139.98, 89.91, 86.84, 74.05, 71.32, 62.12, 21.43; LCMS–ES (*m*/*z*); found [M + H]^+^, 310.13 [C_13_H_19_N_5_O_4_H] requires 310.14. HPLC (reverse phase) 0.5 mL/min MeOH/H_2_O 80:20 in 12 min, λ = 254 nm, Rt = 5.03 min (99%).

#### (2*R*,3*R*,4*S*,5*R*)-2-(6-(Benzylamino)-9*H*-purin-9-yl)-5-(hydroxymethyl)tetrahydrofuran-3,4-diol (**9c**)

Benzylamine (0.46 mL, 4.19 mmol, 1.5 equiv) and TEA (0.58 mL, 4.19 mmol, 1.5 equiv) were added to a stirring solution of 6-chloropurine riboside (800 mg, 2.79 mmol, 1 equiv) in EtOH (15 mL). The reaction was then refluxed at 80 °C for 16 h. The product crashed out of solution when placed in an ice water bath and stirred vigorously. OL031 was then filtered off and dried under reduced pressure to give a white solid (948 mg, 95%); ^1^H NMR (500 MHz, MeOD): δ 8.26 (1H, s), 8.23 (1H, s), 7.38 (2H, d, *J* = 7.6 Hz), 7.31 (2H, t, *J* = 7.5 Hz), 7.24 (1H, t, *J* = 7.3 Hz), 5.96 (1H, d, *J* = 6.5 Hz), 4.75 (1H, dd, *J* = 6.4, 5.1 Hz), 4.32 (1H, dd, *J* = 5.1, 2.5 Hz), 4.17 (1H, q, *J* = 2.5 Hz), 3.90–3.73 (2H, m); ^13^C NMR (126 MHz, MeOD): δ 128.15, 127.11, 126.84, 89.92, 86.83, 74.05, 71.31, 62.12; LCMS–ES (*m*/*z*); found [M + H]^+^, 358.13 [C_17_H_19_N_5_O_4_H] requires 358.14. HPLC (reverse phase) 0.5 mL/min MeOH/H_2_O 80:20 in 12 min, λ = 254 nm, Rt = 5.34 min (99%).

#### (2*R*,3*S*,4*R*,5*R*)-2-(Hydroxymethyl)-5-(6-((4-hydroxyphenethyl)amino)-9*H*-purin-9-yl)tetrahydrofuran-3,4-diol (**9d**)

Inosine (500 mg, 1.86 mmol, 1 equiv), tyramine (384 mg, 2.80 mmol, 1.5 equiv), and PyBOP (1.46 g, 2.80 mmol, 1.5 equiv) were dissolved in anhydrous ACN (20 mL) and substoichiometric amounts of DMF (2 mL) under an inert atmosphere. DIPEA (0.65 mL, 3.73 mmol, 2 equiv) was then added slowly over 5 min at RT, and the reaction was left to stir for 3 days. The solvent was then removed under reduced pressure. The crude oil that remained was then purified by column chromatography using DCM/MeOH (19:1) as an eluent to give a white solid (347 mg, 48%); ^1^H NMR (500 MHz, DMSO): δ 8.34 (1H, s), 8.24 (1H, s), 7.04 (2H, d, *J* = 7.3 Hz), 6.68 (2H, d, *J* = 8.4 Hz), 5.89 (1H, d, *J* = 6.2 Hz), 4.63–4.60 (1H, m), 4.16–4.14 (1H, m), 3.98–3.96 (1H, m), 3.70–3.64 (2H, m) 3.58–3.53 (2H, m), 2.81–2.78 (2H, m); ^13^C NMR (126 MHz, DMSO): δ 129.99, 115.58, 86.37, 73.92, 71.13, 62.14, 36.79; LCMS–ES (*m*/*z*); found [M + H]^+^, 388.20 [C_18_H_21_N_5_O_5_H] requires 388.15. HPLC (reverse phase) 0.5 mL/min MeCN/H_2_O 80:20 in 12 min, λ = 254 nm, Rt = 4.38 min (99%).

#### (2*R*,3*R*,4*S*,5*R*)-2-(6-(Cyclopentylamino)-9*H*-purin-9-yl)-5-(hydroxymethyl)tetrahydrofuran-3,4-diol (**9e**)

Inosine (750 mg, 2.80 mmol, 1 equiv), cyclopentylamine (0.41 mL, 4.19 mmol, 1.5 equiv), and PyBOP (2.183 g, 4.19 mmol, 1.5 equiv) were dissolved in anhydrous ACN (20 mL) and substoichiometric amounts of DMF (2 mL) under an inert atmosphere. DIPEA (0.97 mL, 5.59 mmol, 2 equiv) was then added slowly over 5 min at RT, and the reaction was left to stir for 3 days. The solvent was then removed under reduced pressure. The crude oil that remained was then purified by column chromatography using DCM/MeOH (19:1) as an eluent to give a white solid (749 mg, 80%); ^1^H NMR (500 MHz, MeOD): δ 8.27 (1H, s), 8.23 (1H, s), 5.97 (1H, d, *J* = 6.5 Hz), 4.76 (1H, dd, *J* = 6.4, 5.1 Hz), 4.34 (1H, dd, *J* = 5.1, 2.5 Hz), 4.19 (1H, q, *J* = 2.5 Hz), 3.92–3.75 (2H, m), 2.15–2.09 (1H, m), 1.86–1.61 (8H, s); ^13^C NMR (126 MHz, DMSO): δ 152.84, 140.06, 88.42, 86.37, 73.92, 71.12, 62.14, 32.49, 23.93; LCMS–ES (*m*/*z*); found [M + H]^+^, 336.16 [C_15_H_21_N_5_O_4_H] requires 336.16. HPLC (reverse phase) 0.5 mL/min MeOH/H_2_O 80:20 in 12 min, λ = 254 nm, Rt = 5.43 min (95%).

#### (2*R*,3*S*,4*R*,5*R*)-2-(Hydroxymethyl)-5-(6-(neopentylamino)-9*H*-purin-9-yl)tetrahydrofuran-3,4-diol (**9f**)

Inosine (300 mg, 1.12 mmol, 1 equiv), neopentylamine (0.20 mL, 1.68 mmol, 1.5 equiv), and PyBOP (873 mg, 1.68 mmol, 1.5 equiv) were dissolved in anhydrous ACN (20 mL) and substoichiometric amounts of DMF (2 mL) under an inert atmosphere. DIPEA (0.39 mL, 2.24 mmol, 2 equiv) was then added slowly over 5 min at RT, and the reaction was left to stir for 3 days. The solvent was then removed under reduced pressure. The crude oil that remained was then purified by column chromatography using DCM/MeOH (19:1) as an eluent to give a white solid (305 mg, 81%); ^1^H NMR (500 MHz, MeOD): δ 8.27 (1H, s), 8.20 (1H, s), 5.95 (1H, d, *J* = 6.5 Hz), 4.75 (1H, dd, *J* = 6.4, 5.1 Hz), 4.33 (1H, dd, *J* = 5.1, 2.5 Hz), 4.17 (1H, q, *J* = 2.5 Hz), 3.90–3.73 (2H, m), 3.50–3.46 (2H, m), 1.00 (9H, s); ^13^C NMR (126 MHz, MeOD): δ 155.42, 152.13, 147.66, 140.04, 119.84, 89.94, 86.86, 74.06, 71.33, 62.13, 51.06, 31.94, 26.28; LCMS–ES (*m*/*z*); found [M + H]^+^, 338.18 [C_15_H_23_N_5_O_4_H] requires 338.18. HPLC (reverse phase) 0.5 mL/min MeCN/H_2_O 80:20 in 12 min, λ = 254 nm, Rt = 4.66 min (99%).

### Cell Culture

Both HeLa and Parkin-overexpressed HeLa cells were maintained in DMEM high glucose (Gibco) and 10% FBS (Sigma-Aldrich) at 37 °C with 5% CO_2_. For experiments, cells were counted and seeded into a range of culture plates, and depending on the experiment, cell counting was done using the Cellometer Auto T4 with trypan blue (Gibco). HeLa cells were incubated at 37 °C in 5% CO_2_ in a T75 flask (Corning) until a confluency of 70–80% was achieved at which point the cells were used in an experiment. Alternatively, HeLa cells were seeded in 6-well plates and then transfected with 0.2 μg mL^–1^ Parkin cDNA using the PEI method once the plated cells reached 60% confluency. After 6 h, the media was changed. Sub-culturing was done when the cells reached ∼90% confluency.

### Preparation of Total Protein Lysate and Protein Concentration Measurement

Cells at 70–80% confluency were lysed as follows: cells were washed with phosphate-buffered saline (PBS) (Sigma). 150 μL of lysis buffer was used on each well containing either (1) 50 mM Tris–HCl pH 7.5, 1 mM EDTA, 1 mM EGTA, 0.27 M sucrose, 1 mM Na_3_VO_4_, 50 mM NaF, 5 mM Na pyrophosphate and fresh 1 mM benzamidine, 1% NP-40, and 0.1 mM PMSF or (2) 50 mM Tris–HCl pH 7.5, 1 mM EDTA, 1 mM EGTA, 10 mM Na β-glycerophosphate, 0.27 M sucrose, 1 mM Na_3_VO_4_, 50 mM NaF, 10 mM Na pyrophosphate with fresh 1 mM benzamidine, 1% Triton X-100, complete EDTA-free protease inhibitor, phosphatase inhibitor cocktail 3, and 100 μM 2-chloroacetamide. The cells were scrapped and transferred to the microtube and eventually spun down at 12,000 rpm for 15 min at 4 °C. Finally, the supernatant was transferred to the new microtubes and stored at −20 °C. Protein concentration was measured using Bradford assay. Serial concentrations 0.125, 0.25, 0.5, and 1 mg mL^–1^ of bovine serum albumin (BSA) (Sigma-Aldrich) were used as a standard. Samples were boiled at 90 °C for 5 min in SDS sample loading buffer.

### Antibodies

Anti-GAPDH (1:1000 5% BSA/TBS-T, Cell Signaling), anti-Parkin phospho-Ser65 (2 μg/mL, 5% milk/TBS-T, S210D, second bleed, University of Dundee), anti-Parkin phospho-Ser65 (2 μg/mL, 5% milk/TBS-T, S210D, third bleed, University of Dundee), anti-pParkin total (2 μg/mL, 5% milk/TBS-T, S966C, second bleed, University of Dundee), anti-Parkin phospho-Ser65 (1:10,000 in 5% BSA/TBS-T, rabbit monoclonal, MJF foundation), non-phosphopeptide Parkin Ser65 (2 mg/mL, 5% milk/TBS-T, University of Dundee), anti-PINK1 total (2 μg/mL, 5% milk/TBS-T, S085D, third bleed, University of Dundee), anti-Bcl-xL total (1:1000, 5% BSA/TBS-T, Cell Signaling), anti-Bcl-xL phospho-Ser62 (1:1000, 5% BSA/TBS-T, Invitrogen), anti-PINK1 phospho-Thr257 (2 μg/mL, 5% milk/TBS-T, S114D, third bleed, University of Dundee), non-phosphopeptide PINK1 Thr257 (2 mg/mL, 5% milk/TBS-T, University of Dundee), anti-OPA1 (1:1000, 5% BSA/TBS-T, BD Biosciences), anti-rabbit IgG HRP-linked (1:1000 5% BSA/TBS-T, Cell Signaling), anti-sheep IgG HRP-linked (1:5000 5% milk/TBS-T, Abcam), and anti-mouse IgG HRP-linked (1:1000 5% BSA/TBS-T, Cell Signaling) were used.

### Phosphorylated Ubiquitin Immunoblotting

Cells were pre-treated with KR for 23 h followed by 1 h treatment of 10 μM CCCP or niclosamide. Cells were then harvested using AP lysis buffer [50 mM Tris–HCl (pH 7.5), 50 mM NaCl, 1% IGEPAL, 20 mM MgCl_2_, 5 mM 2-mercaptoethanol, 10% glycerol (v/v), 1× protease inhibitor cocktail (Roche), and 1× phosphatase inhibitor cocktail (Roche)]. Denatured protein was loaded in Mini-PROTEAN TGX 4–12% precast gels, followed by protein separation. Proteins were then transferred to a methanol-activated PVDF membrane using the Trans-Blot Turbo system (Bio-Rad), and membranes were subsequently blocked with 5% non-fat dry milk in TBS/0.1% Tween (TBST) for 1 h at room temperature. Following blocking, membranes were washed in TBST and were incubated in primary antibodies diluted in 5% BSA/TBST or 5% milk/TSBT overnight at 4 °C with agitation. The next day, membranes were washed in TBST and incubated with horse radish peroxidase (HRP)-conjugated antibodies for 1 h at room temperature with agitation. Following TBST washes, proteins were visualized using an Amersham ECL Prime kit (GE Biosciences) and imaged using the Bio-Rad ChemicDoc. Primary antibodies used for western blotting: GAPDH (Abcam cat #ab9485), phospho-Ub Ser 65 (Sigma-Aldrich cat #ABS1513-I), and LC3B (Cell Signaling cat #3868S). Secondary antibodies used for western blotting: anti-rabbit HRP (Agilent Dako cat #P0399) and anti-mouse HRP (Agilent Dako cat #P0260).

### Immunofluorescence

Cells were seeded on PerkinElmer PhenoPlate 96-well plates, followed by treatment with compounds. Cells were fixed in chilled 4% PFA for 10 min followed by washes in PBS. Permeabilization of cells was achieved with the addition of PBS/0.1% Triton X-100 for 5 min at room temperature. Following PBS washes, cells were blocked using blocking buffer (PBS, 5% BSA, 0.1% Tween 20) for 1 h at room temperature. Primary antibodies were diluted in blocking buffer and added to cells to be incubated overnight at 4 °C. Cells were subsequently washed with PBS-T, and the appropriate secondary antibodies (diluted in blocking buffer) were added for 2 h at room temperature in darkness. 10 μg/mL of Hoechst 33258 stain was added with secondary antibodies when required. Cells were again washed with PBS-T followed by the addition of PBS with 0.02% sodium azide. Cells were then stored at 4 °C until ready for imaging. Primary antibodies used for immunofluorescence are as follows: phospho-Ub Ser 65 (Sigma-Aldrich cat #ABS1513-I) and TOM20 (Santa-Cruz cat #sc-17764). Secondary antibodies and stains used in immunofluorescence: anti-IgG2a Alexa Fluor 546 (Thermo Fisher cat #A21133), anti-rabbit Alexa Fluor 647 (Thermo Fisher cat #A21244), and Hoechst 33258 stain (Sigma-Aldrich cat #B2883).

Confocal microscopy was performed using a Zeiss LSM800 w/Airyscan fluorescent microscope equipped with lasers emitting at 405, 488, 561, and 640 nm. All images were captured using a 40× oil immersion objective lens and processed using Zeiss ZEN software. Images were analyzed and quantified using Columbus (PerkinElmer) and TIBCO Spotfire software.

For assessing tetramethylrhodamine methyl ester perchlorate (TMRM) fluorescence, cell media was removed and replaced with cell media consisting of 5 nM TMRM (Thermo Fisher) and 10 μg/mL Hoechst 33258 stain for 30 min in the dark at 37 °C to allow incorporation of TMRM into mitochondria. Following PBS washes, FluoroBrite DMEM (Gibco) media was added to cells, followed by subsequent imaging using a Zeiss LSM800 microscope at 37 °C with 5% CO_2_.

### ELISA Quantification of pS65-Ub in Primary Mouse Astrocytes

Astrocytes were derived from postnatal day 3 mouse pups and cultured essentially as described.^1^ Briefly, C57BL/6J mouse pups were decapitated, and the dissected cortices (with meninges removed) were incubated with 0.25% trypsin at 37 °C for 10 min; then, the trypsin was replaced with astrocyte media consisting of Dulbecco’s modified Eagle’s medium with 10% fetal bovine serum and penicillin/streptomycin antibiotics. The tissue was triturated using a 5 mL plastic pipette; then, the dissociated cells were filtered through a 100 μm filter and incubated at 37 °C in 12-well tissue culture plates, changing the media every 3–4 days. When astrocytes were 70–90% confluent (7–10 days in vitro), cells were treated with the compounds described or DMSO control for 24 h, with the addition of 10 nM valinomycin or DMSO control for the final 5 h. After aspirating the media and briefly washing with PBS, cells were lysed by pipetting up and down in ice cold lysis buffer consisting of 20 mM HEPES, pH 8.0, 10 mM NaCl, 3 mM MgCl_2_, and 0.1% NP-40 detergent supplemented with a cocktail of protease and phosphatase inhibitors (MCE HY-K0010, HY-K0022, and HY-K0023). The cell lysates were centrifuged at 12,000*g* for 10 min at 4 °C; then, the pellets were resuspended in RIPA buffer, centrifuged again at 12,000*g* for 10 min at 4 °C, and then analyzed by pS65-Ub ELISA essentially as described.^2^

### Mitophagy Assay

#### Flow Cytometry Analysis

2.5 × 10^5^ cells were seeded in a 6 cm dish the day before. After treatment, cells were washed with PBS, trypsinized for 5 min, and centrifuged 3 min at 1200 rpm. The pellet of cells was resuspended in 250 μL of PBS and 1 mL of 3.7% (w/v) formaldehyde, and 200 mM HEPES pH 7.0 was added. After 30 min at RT, 2 mL of PBS was added before centrifugation 5 min at 1200 rpm. Finally, the pellet of cells was resuspended in 1% FCS in PBS and analyzed by flow cytometry. For each independent experiment, at least 2 × 10^4^ cells were acquired on an LSRFortessa cell analyzer. Based on FCS and SSC profiles, living cells were gated. As negative control, cells expressing any mitophagy reporter were used. To quantify the percentage of cells undergoing mitophagy, the ratio GFP/mCherry was analyzed. The gate used for the untreated condition or control cells was applied to all the other conditions. GraphPad Prism software was used for statistical analysis. Significance was determined by two-way ANOVA with Sidak’s multiple comparisons test. *P*-values are indicated as *P < 0.05.

#### Microscopy

Cells stably expressing the *mito*-QC mitophagy reporter system (mCherry-GFP-FIS1^101-152^) were seeded onto sterile glass coverslips in 24-well dishes. After treatment, coverslips were washed once with PBS, fixed with 3.7% (w/v) formaldehyde and 200 mM HEPES pH 7.0 for 10 min, and washed twice with PBS. After nuclear counterstaining with 1 μg/mL Hoechst-33258 dye, slides were washed and mounted in ProLong Gold (Invitrogen). Observations were made with a Zeiss LSM880 Airyscan confocal scanning microscope (ZEISS, 63X objective, NA 1.4). Images were processed using ImageJ and Adobe Photoshop software.

## Supporting Information Available

Molecular formula strings (CSV)
Illustrations showing that kinetin riboside does not prevent the membrane potential collapse caused by niclosamide and CCCP, kinetin riboside and nucleosides 9a and 9c do not impact mitochondrial fragmentation, Parkin localization, or ubiquitin phosphorylation, docking of kinetin riboside and nucleoside 9c, effect of kinetin riboside, nucleoside 9c, and its triphosphate derivative on *Tc*PINK1 in vitro, in vitro kinase assay, protein sequence alignment, superimposition of the full-length AlphaFold human PINK1 and full-length *Tc*PINK1, in silico docking, in vitro kinase assay methods, and HPLC chromatograms of the final compounds (PDF)

## Abbreviations

CCCP: carbonyl cyanide *m*-chlorophenyl-hydrazine
DIPEA: *N*,*N*-diisopropylethylamine
DMF: dimethylformamide
FCCP: carbonyl cyanide *p*-(trifluoromethoxy)phenyl-hydrazone
KO: knock-out
KR: kinetin riboside
OPA1: optic atrophy protein 1
PD: Parkinson’s disease
PINK1: PTEN-induced kinase 1
PyBOP: benzotriazol-1-yloxytripyrrolidinophosphonium hexafluorophosphate
