# Supplementary material for: PINK1-Dependent Mitophagy Inhibits Elevated Ubiquitin Phosphorylation Caused by Mitochondrial Damage
Source: J Med Chem. 2023 May 30;66(11):7645–56. doi: 10.1021/acs.jmedchem.3c00555 (PMC10258795; doi:10.1021/acs.jmedchem.3c00555)
Supplement: Supplementary file 2 — jm3c00555_si_002.pdf [file jm3c00555_si_002.pdf]

Supporting Information

PINK1-Dependent Mitophagy Inhibits Elevated Ubiquitin Phosphorylation Caused by Mitochondrial Damage

Olivia A. Lambourne,[1] Shane Bell,[2] Léa P. Wilhelm,[3] Erika B. Yarbrough,[4] Gabriel G. Holly,[4] Oliver M. Russell,[2] Arwa M. Alghamdi,[1] Azeza M. Fdel,[1] Carmine Varricchio,[1] Emma L. Lane,[1] Ian G. Ganley,[3] Arwyn T. Jones,[1] Matthew S. Goldberg,[4] Youcef Mehellou[1,*]

[1]*Cardiff School of Pharmacy and Pharmaceutical Sciences, Cardiff University, Cardiff CF10 3NB, U.K.*
[2]*Wellcome Centre for Mitochondrial Research, Newcastle University, Newcastle upon Tyne NE2 4HH, U.K.*
[3]*MRC Protein Phosphorylation and Ubiquitylation Unit, University of Dundee, Dundee, U.K.*
[4]*Center for Neurodegeneration and Experimental Therapeutics, Department of Neurology, The University of Alabama at Birmingham, Birmingham, AL, 35294, USA.*

Content:

I. Supporting Figures……………………………………………………………………………………S2-S8
II. Methods……………………………………………………………………………………………S9
III. HPLC Chromatograms of the Final Compounds……………………………………………S10-S21

I. SUPPORTING FIGURES

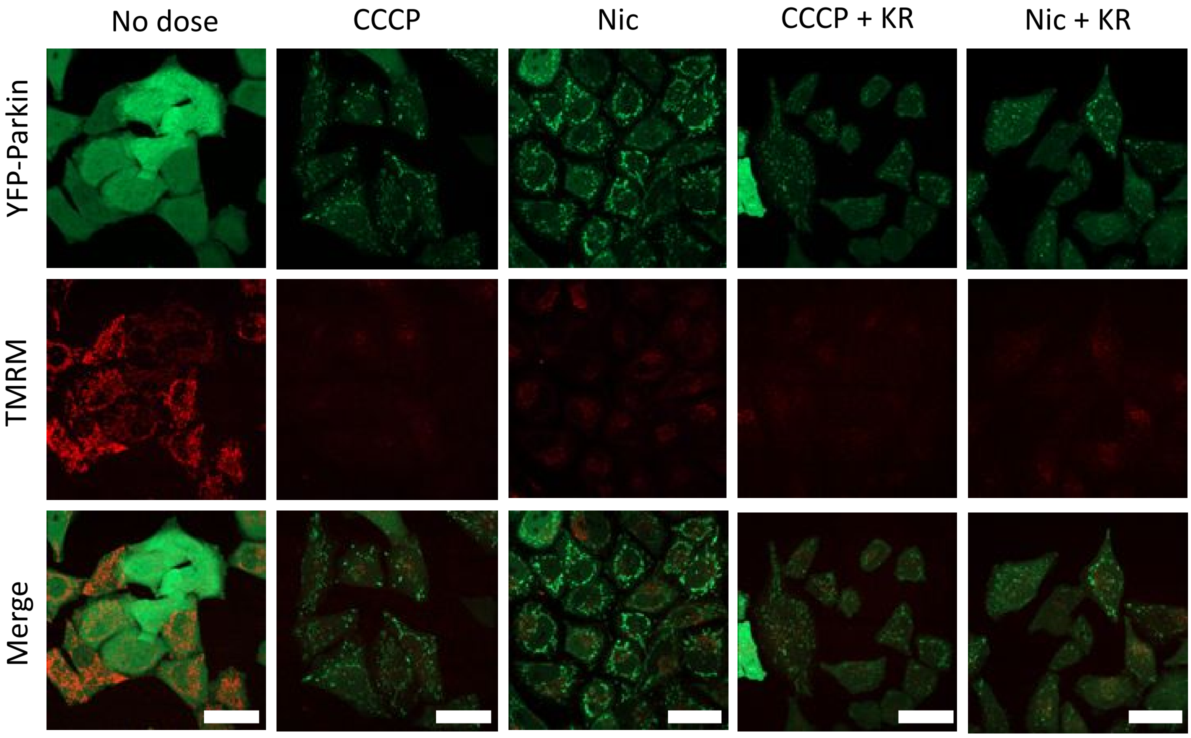

Supporting Figure S1. kinetin riboside does not prevent the membrane potential collapse caused by niclosamide and CCCP. HeLa cells transfected with YFP-parkin were pre-treated with 50 µM kinetin riboside for 24 h followed by treatment with 10 µM CCCP or niclosamide for 1 h. Cells were treated with 5nM Tetramethylrhodamine, methyl ester (TMRM), followed by confocal imaging of TMRM and YFP-Parkin expression using a Zeiss LSM800 microscope. Scale bar = 40 µm.

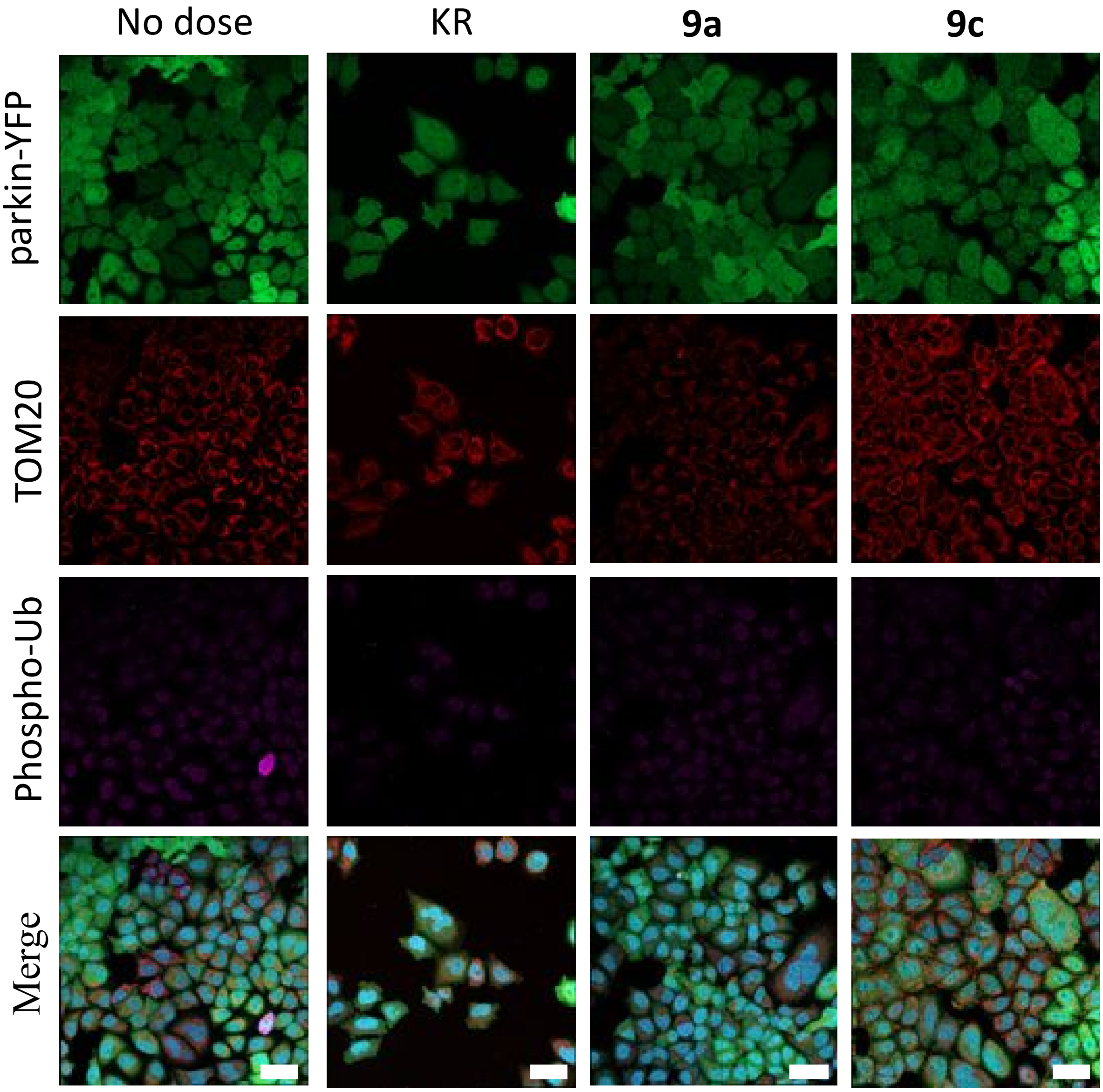

Supporting Figure S2. Kinetin riboside and nucleosides 9a and 9c do not impact mitochondrial fragmentation, parkin localisation or ubiquitin phosphorylation. HeLa cells transfected with YFP-parkin were pre-treated with 50 µM kinetin riboside or *N*^{6}-benzyladenosine for 24 h followed by fixation with PFA. Immunofluorescence was performed probing for Ub Ser65 phosphorylation (pUb), YFP-parkin expression, and mitochondrial mass (TOM20) and imaged using the Zeiss LSM800 confocal microscope. Scale bar = 40 µm.

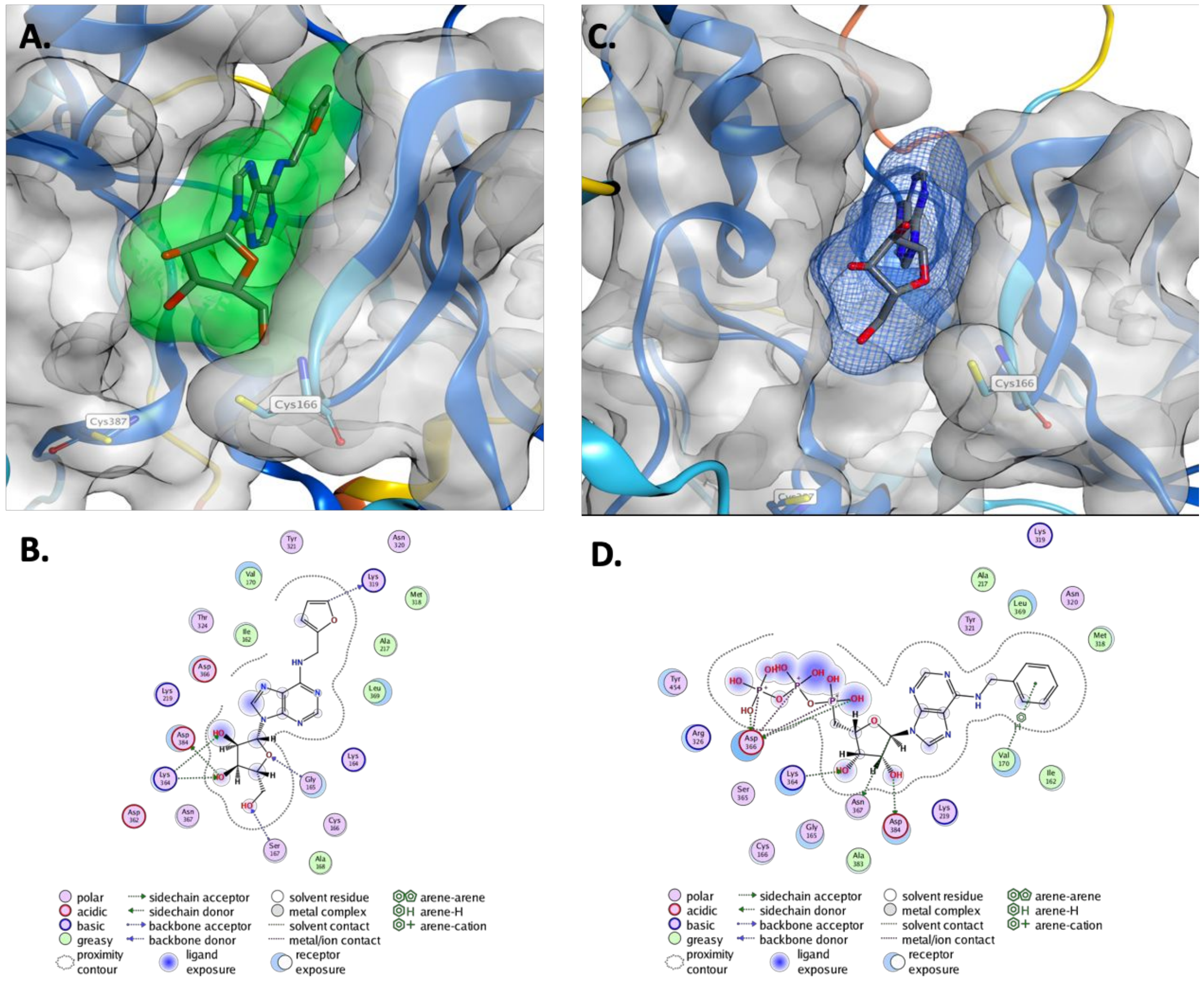

Supporting Figure S3. Docking of kinetin riboside and nucleoside 9c into the human AlphaFold structure of human PINK1. A. Docking of kinetin riboside into human PINK1. B. 2D interactions between kinetin riboside and human PINK1. C. Docking of nucleoside 9c into human PINK1. 2D interactions between nucleoside 9c and human PINK1.

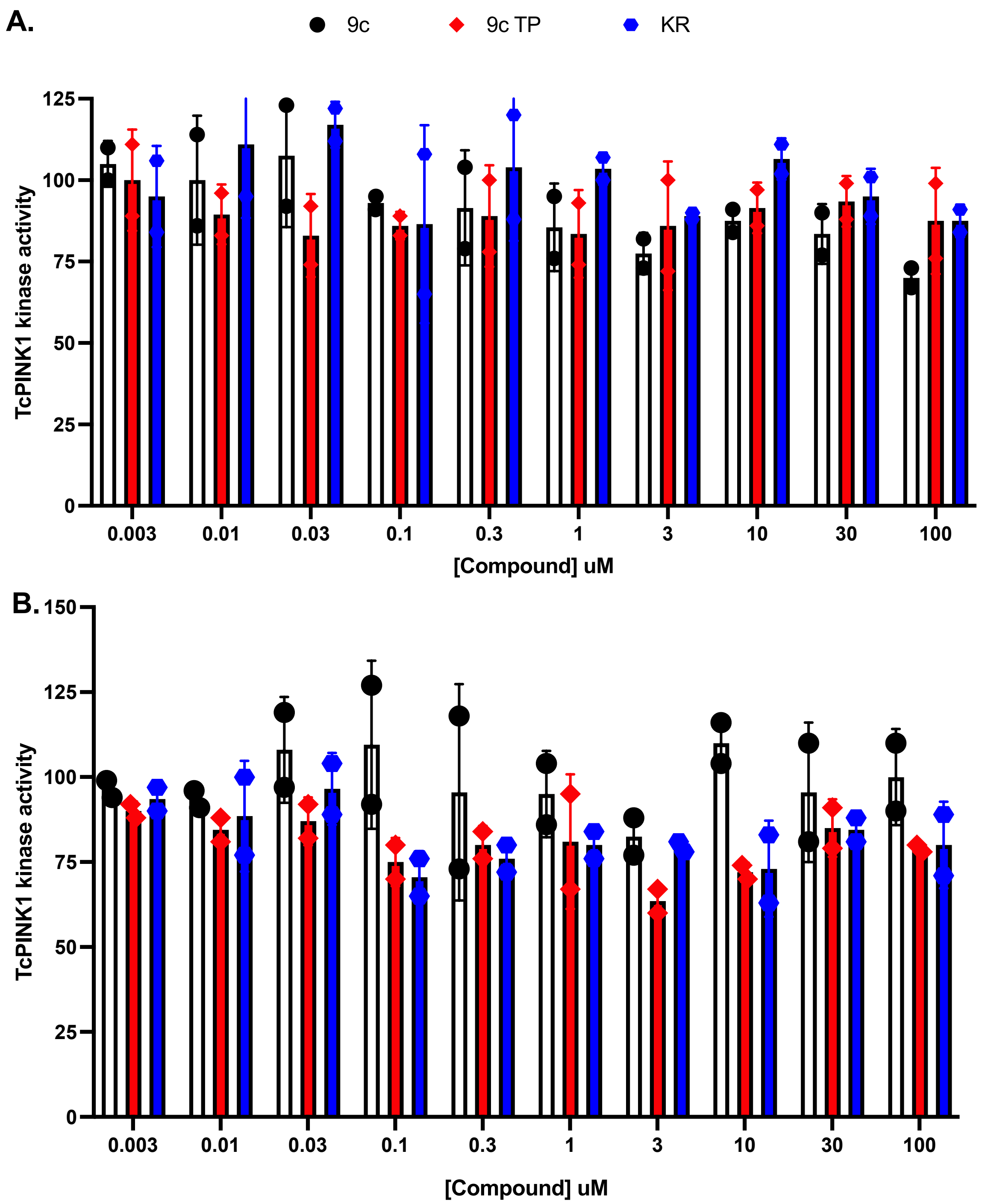

Supporting Figure S4. Effect of kinetin riboside, nucleoside 9c and its triphosphate derivative on TcPINK1 in vitro employing either ubiquitin (A) or parkin (B) as substrates.

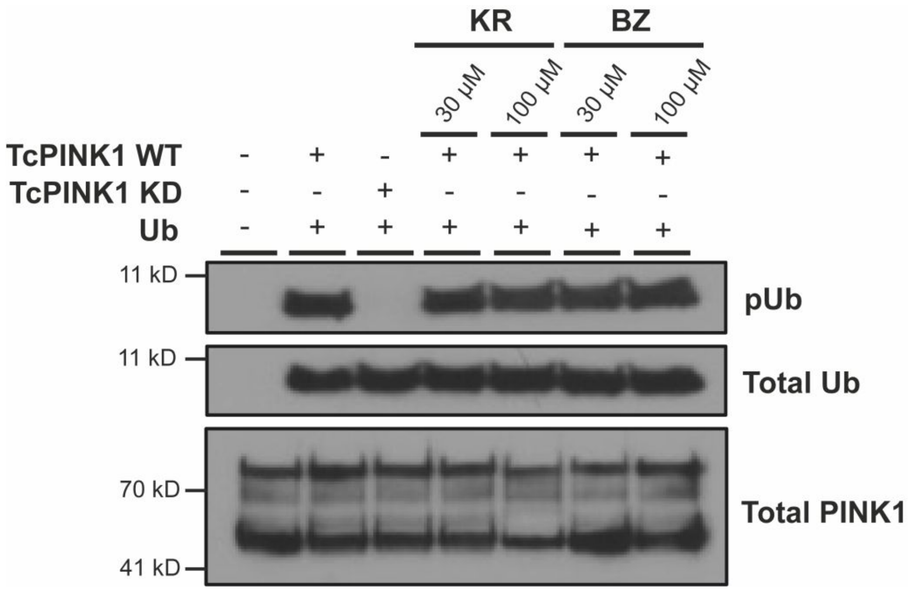

Supporting Figure S5. In vitro kinase assay examining the effects of kinetin riboside and *N*[6]-benzyladenosine on the PINK1 kinase activity. Kinase reactions employed 1 µg of either recombinant MBP-tagged wild-type or kinase-dead (D359A) TcPINK1, 1.5 µg of recombinant human ubiquitin, , 0.1 mM ATP, 10 mM EGTA, 50 mM Tris-HCl (pH 7.5), 0.2 mM DTT, buffer A, and 30 µM or 100 µM of Kinetin riboside (KR) or *N*[6]-benzyladenosine (BZ). Total reaction volume was 25 µL, and the total DMSO volume was 5%. The reaction was carried out for 45 minutes at 30 ˚C and terminated by addition of SDS loading buffer and separate by SDS-PAGE. The samples underwent Western blotting for total PINK1, total ubiquitin and ubiquitin pSer65.

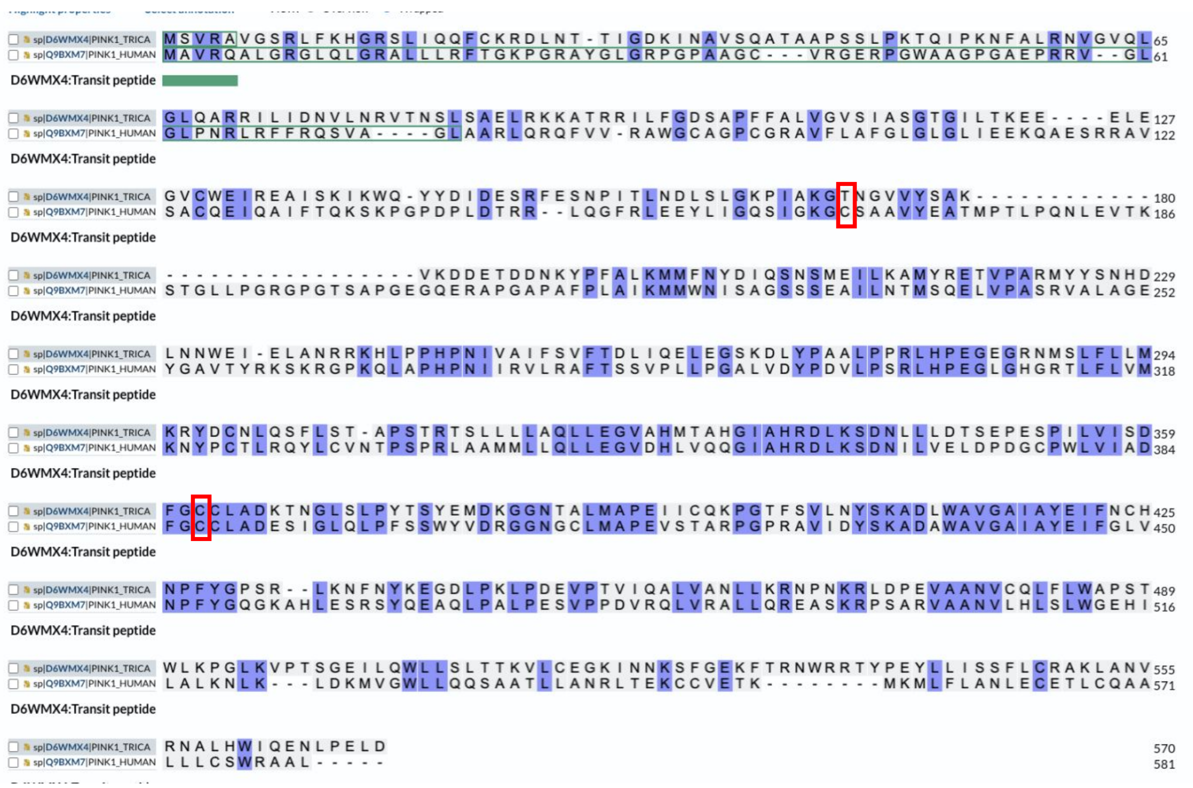

Supporting Figure S6. Protein sequence alignment of human PINK1 [hPINK1] (Q9BXM7) and *Tribolium castaneum* PINK1 [TcPINK1] (UniProt code: D6WMX4). The data was obtained using UniProt Align. Human PINK1 Cys166 (equivalent to TcPINK1 T172) and Cys387 (equivalent to TcPINK1 Cys362) are identified in red rectangles. The data shows hPINK1 Cys166 not to be conserved while hPINK1 Cys387 is conserved.

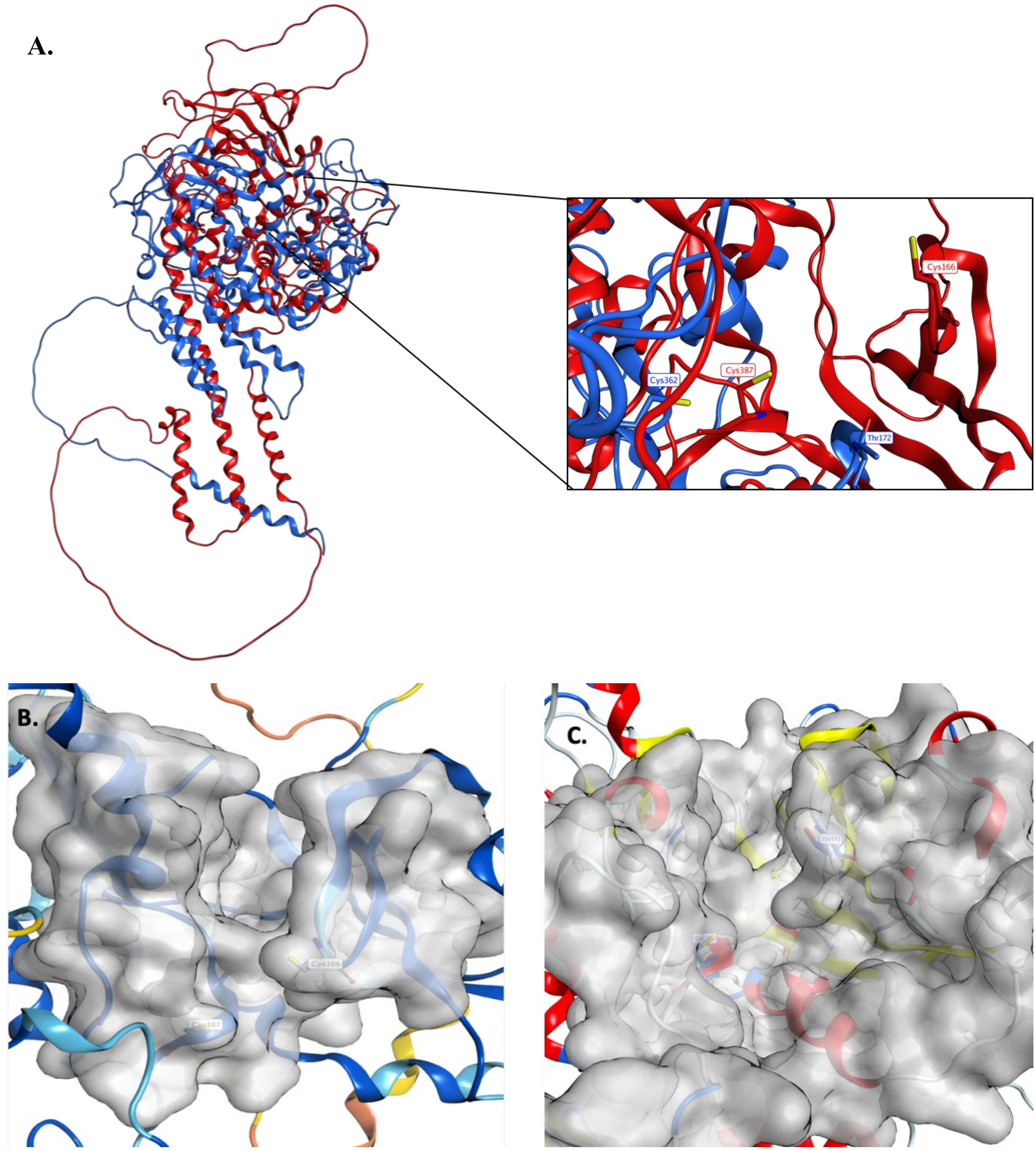

Supporting Figure S7. A. Superimposition of the full length AlphaFold human PINK1 [hPINK1, shown in red] (Q9BXM7) and full length *Tribolium castaneum* PINK1 [TcPINK1, shown in blue] (UniProt code: D6WMX4). ATP binding pocket of B. hPINK1 and C. TcPINK1.

II. METHODS

PINK1 in vitro kinase assay. This assay was carried out by the MRC PPU Reagents and Services (University of Dundee, U.K.). The assay was conducted as follows: TcPINK1 (5-20mU diluted in 50 mM Tris pH 7.5, 0.1 mM EGTA, 1 mg/ml BSA, 0.1% mercaptoethanol) is assayed against GST PARK2 TV3 or Ubiquitin-His in a final volume of 25.5 µl containing 50mM Tris pH 7.5, 0.1mM EDTA, 10mM DTT, protein substrate (0.3mg/ml of GST PARK2 TV3 or 1mg/ml of Ubiquitin-His, 10 mM magnesium acetate and 0.1 mM [^{33}P-γ-ATP] (50-1000 cpm/pmole) in the presence of the relevant small molecule (serial dilution with the highest concentration being 100 µM) and incubated for 30 min at room temperature. Assays are stopped by addition of 5 µl of 0.5 M (3%) orthophosphoric acid and then harvested onto P81 Unifilter plates with a wash buffer of 50 mM orthophosphoric acid.

Molecular docking studies. The human and Tribolium castaneum PINK1 protein models, Q9BXM7 and D6WMX4 respectively, were downloaded from the AlphaFold Protein Structure Database (https://alphafold.ebi.ac.uk/) and prepared using the QuickPrep module in MOE. Predicted protonation states of protein residues were calculated considering a temperature of 300 K and a pH of 7.4. SiteFinder module in MOE was used to define the binding pocket in the proximity of the conserved cys169 residue. The docking studies were performed using the Schrodinger platform. A 12 Å docking grid was prepared using as centroid the cys169 residues and the docking was carried out using Glide SP precision keeping the default parameters and setting. MOE was used to visualize the protein-ligand interaction and acquire the images.

III. HPLC CHROMATOGRAMS

Analysis Report

Compound **8a**

<Sample Information>

Sample Name	: YM-IW5-2
Sample ID	: YM-IW5-80MeOH20H20-2
Data Filename	: YM-IW5-2.lcd
Method Filename	: method 0.5mlmin 251018.lcm
Batch Filename	: YM-IW5-80MeOH20H2O-2.lcb
Vial #	: 1-28
Injection Volume	: 10 uL
Date Acquired	: 14/01/2019 13:57:56
Date Processed	: 14/01/2019 14:09:57
Sample Type	: Unknown
Acquired by	: System Administrator
Processed by	: System Administrator

<Chromatogram>

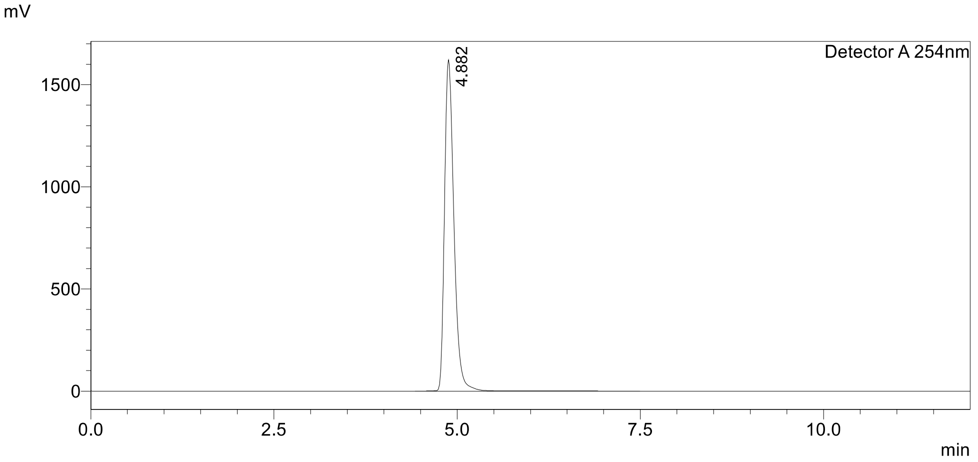

<Peak Table>

Detector A 254nm

Peak#	Ret. Time	Area	Height	Area%
1	4.882	14506907	1621327	100.000
Total		14506907	1621327	100.000

C:\LabSolutions\Data\Project1\YM-IW5-2.lcd

Analysis Report

<Sample Information>

Compound **8b**

Sample Name	: YM-IW4-2
Sample ID	: YM-IW4-90MeOH10H20-1
Data Filename	: YM-IW4-2.lcd
Method Filename	: method 0.5mlmin 251018.lcm
Batch Filename	: YM-IW4-90MeOH10H2O-1.lcb
Vial #	: 1-28
Injection Volume	: 10 uL
Date Acquired	: 16/01/2019 16:34:50
Date Processed	: 16/01/2019 16:46:50

Sample Type	: Unknown
Acquired by	: System Administrator
Processed by	: System Administrator

<Chromatogram>

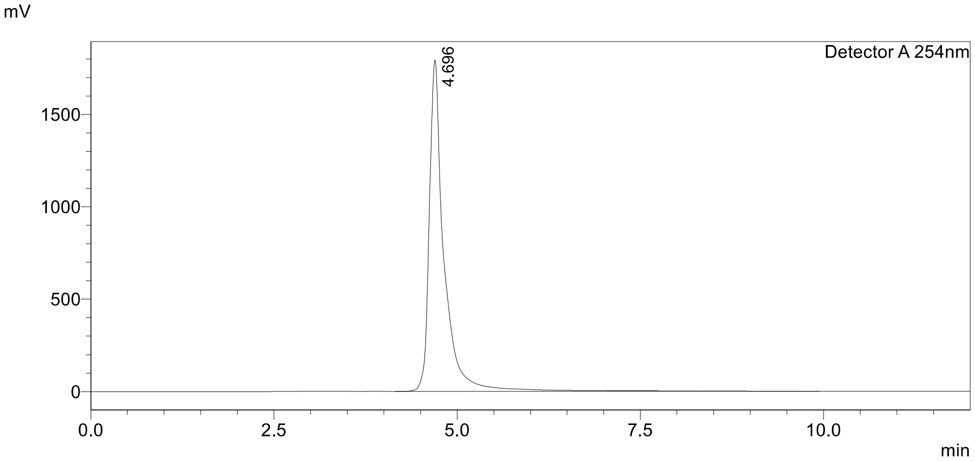

<Peak Table>

Detector A 254nm

Peak#	Ret. Time	Area	Height	Area%
1	4.696	25332865	1793551	100.000
Total		25332865	1793551	100.000

Analysis Report

<Sample Information>

Compound **8c**

Sample Name	: YM-OL035-3
Sample ID	: YM-OL035-80MeOH20H20-3
Data Filename	: YM-OL035-3.lcd
Method Filename	: method 0.5mlmin 251018.lcm
Batch Filename	: YM-OL035-80MeOH20H2O-3.lcb
Vial #	: 1-28
Injection Volume	: 10 uL
Date Acquired	: 07/01/2019 16:22:10
Date Processed	: 07/01/2019 16:34:11
Sample Type	: Unknown
Acquired by	: System Administrator
Processed by	: System Administrator

<Chromatogram>

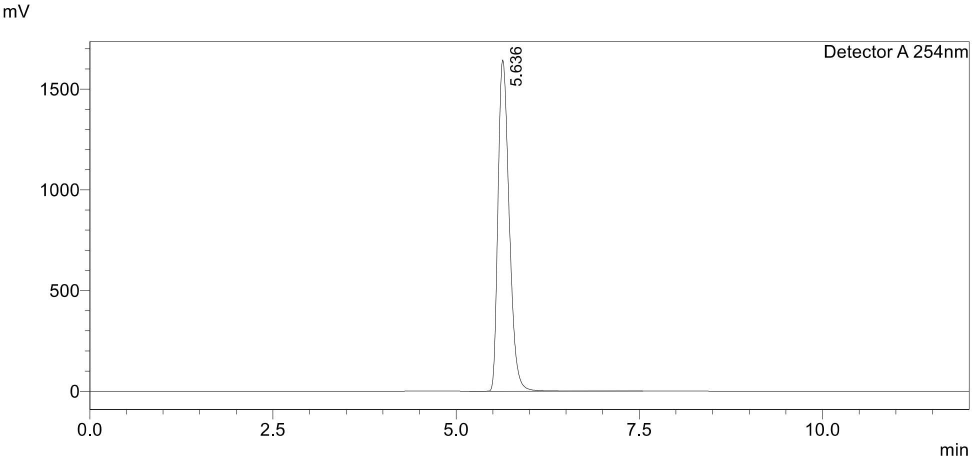

<Peak Table>

Detector A 254nm

Peak#	Ret. Time	Area	Height	Area%
1	5.636	17532595	1644068	100.000
Total		17532595	1644068	100.000

Analysis Report

Compound **8d**

<Sample Information>

Sample Name	: YM-OL037-3		
Sample ID	: YM-OL037-80MeOH20H20-3		
Data Filename	: YM-OL037-3.lcd		
Method Filename	: method 0.5mlmin 251018.lcm		
Batch Filename	: YM-OL037-80MeOH20H2O-3.lcb		
Vial #	: 1-28	Sample Type	: Unknown
Injection Volume	: 10 uL		
Date Acquired	: 14/01/2019 10:22:09	Acquired by	: System Administrator
Date Processed	: 14/01/2019 10:34:11	Processed by	: System Administrator

<Chromatogram>

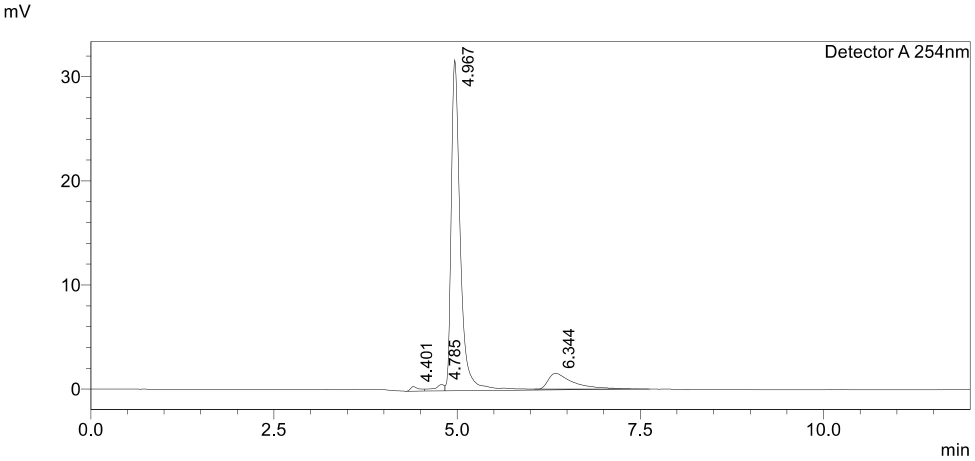

<Peak Table>

Detector A 254nm

Peak#	Ret. Time	Area	Height	Area%
1	4.401	3469	430	1.063
2	4.785	5807	611	1.779
3	4.967	280676	31772	85.991
4	6.344	36451	1517	11.168
Total		326402	34329	100.000

C:\LabSolutions\Data\Project1\YM-OL037-3.lcd

Analysis Report

<Sample Information>

Compound **8e**

Sample Name	: YM-OL036-2		
Sample ID	: YM-OL036-80MeOH20H20-2		
Data Filename	: YM-OL036-2.lcd		
Method Filename	: method 0.5mlmin 251018.lcm		
Batch Filename	: YM-OL036-80MeOH20H20.lcb		
Vial #	: 1-28	Sample Type	: Unknown
Injection Volume	: 10 uL		
Date Acquired	: 07/01/2019 11:32:17	Acquired by	: System Administrator
Date Processed	: 07/01/2019 11:44:17	Processed by	: System Administrator

<Chromatogram>

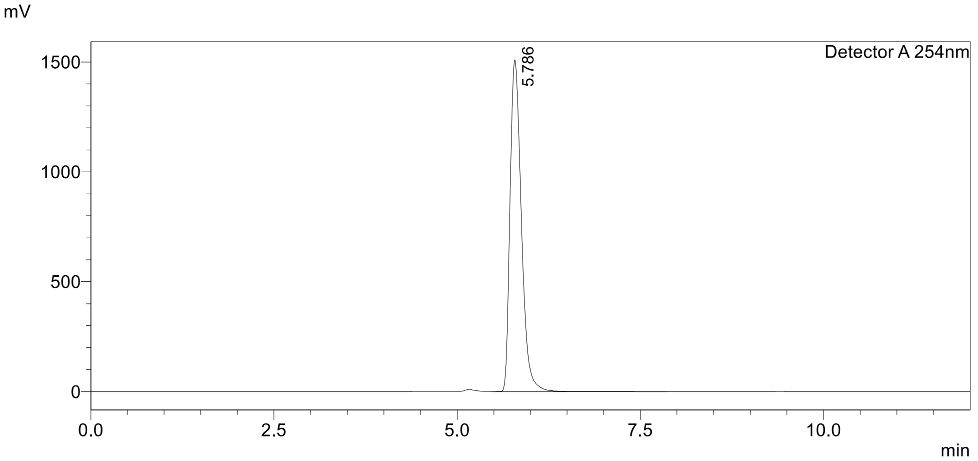

<Peak Table>

Detector A 254nm

Peak#	Ret. Time	Area	Height	Area%
1	5.786	16195268	1508348	100.000
Total		16195268	1508348	100.000

C:\LabSolutions\Data\YM\AM\YM-OL036-2.lcd

Analysis Report

<Sample Information>

Compound **8f**

Sample Name	: YM-OL034-2
Sample ID	: YM-OL034-90MeOH10H20-2
Data Filename	: YM-OL034-2.lcd
Method Filename	: method 0.5mlmin 251018.lcm
Batch Filename	: YM-OL034-90MeOH10H2O-2.lcb
Vial #	: 1-28
Injection Volume	: 10 uL
Date Acquired	: 17/01/2019 10:30:56
Date Processed	: 17/01/2019 10:42:57
Sample Type	: Unknown
Acquired by	: System Administrator
Processed by	: System Administrator

<Chromatogram>

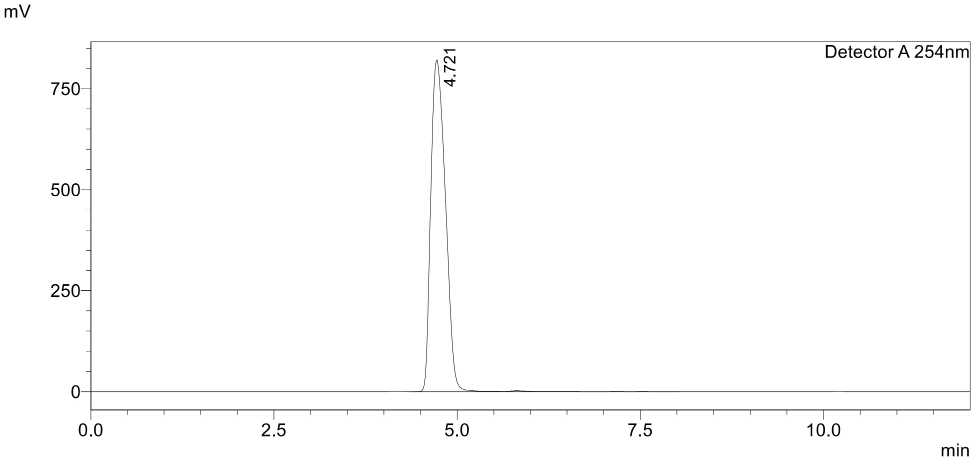

<Peak Table>

Detector A 254nm

Peak#	Ret. Time	Area	Height	Area%
1	4.721	11425380	820890	100.000
Total		11425380	820890	100.000

Analysis Report

<Sample Information>

Compound **9a**

Sample Name	: YM-IW3-5
Sample ID	: YM-IW3-90MeOH10H20-1
Data Filename	: YM-IW3-5.lcd
Method Filename	: method 0.5mlmin 251018.lcm
Batch Filename	: YM-IW3-90MeOH10H2O-1.lcb
Vial #	: 1-28
Injection Volume	: 10 uL
Date Acquired	: 17/01/2019 10:11:43
Date Processed	: 17/01/2019 10:23:45
Sample Type	: Unknown
Acquired by	: System Administrator
Processed by	: System Administrator

<Chromatogram>

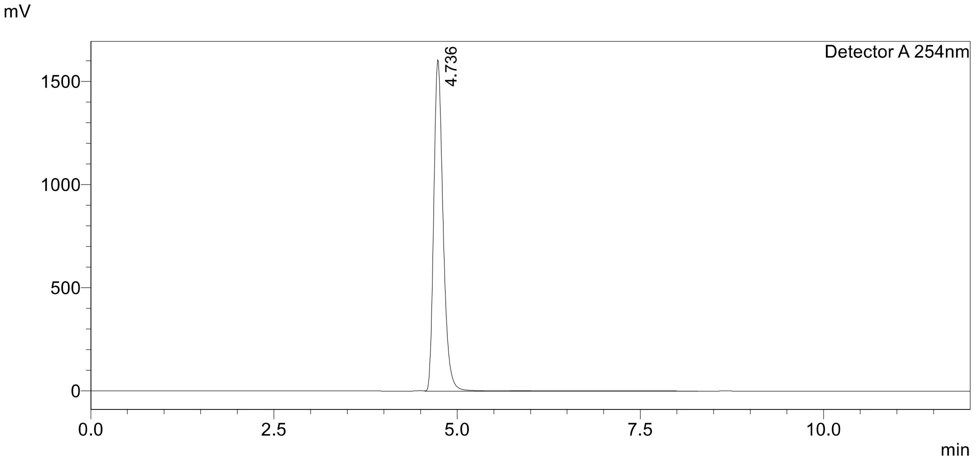

<Peak Table>

Detector A 254nm

Peak#	Ret. Time	Area	Height	Area%
1	4.736	14355239	1604807	100.000
Total		14355239	1604807	100.000

Analysis Report

<Sample Information>

Compound **9b**

Sample Name	: YM-IW2-3
Sample ID	: YM-IW2-80MeOH20H20-3
Data Filename	: YM-IW2-3.lcd
Method Filename	: method 0.5mlmin 251018.lcm
Batch Filename	: YM-IW2-80MeOH20H2O-3.lcb
Vial #	: 1-28
Injection Volume	: 10 uL
Date Acquired	: 08/01/2019 16:03:59
Date Processed	: 08/01/2019 16:16:00
Sample Type	: Unknown
Acquired by	: System Administrator
Processed by	: System Administrator

<Chromatogram>

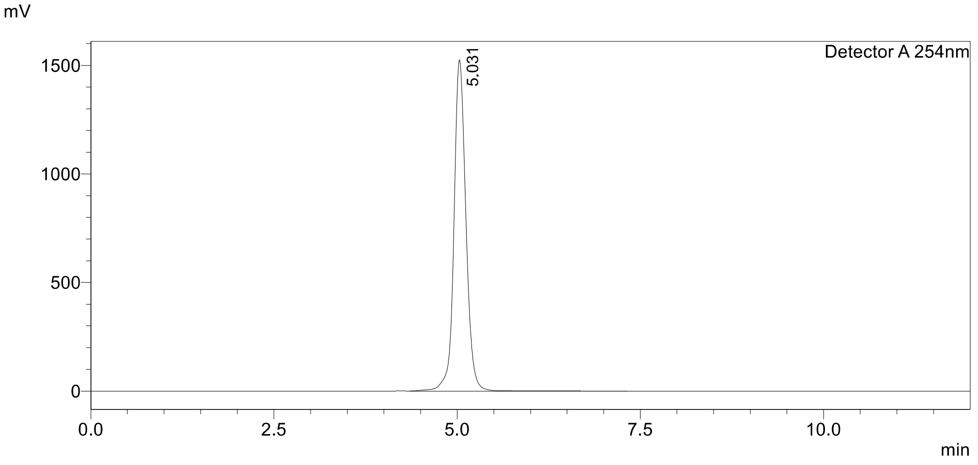

<Peak Table>

Detector A 254nm

Peak#	Ret. Time	Area	Height	Area%
1	5.031	17453511	1525060	100.000
Total		17453511	1525060	100.000

C:\LabSolutions\Data\Project1\YM-IW2-3.lcd

Analysis Report

<Sample Information>

Compound **9c**

Sample Name	: YM-OL031-3		
Sample ID	: YM-OL031-80MeOH20H20-3		
Data Filename	: YM-OL031-3.lcd		
Method Filename	: method 0.5mlmin 251018.lcm		
Batch Filename	: YM-OL031-80MeOH20H2O-3.lcb		
Vial #	: 1-28	Sample Type	: Unknown
Injection Volume	: 10 uL		
Date Acquired	: 16/01/2019 16:02:44	Acquired by	: System Administrator
Date Processed	: 16/01/2019 16:14:46	Processed by	: System Administrator

<Chromatogram>

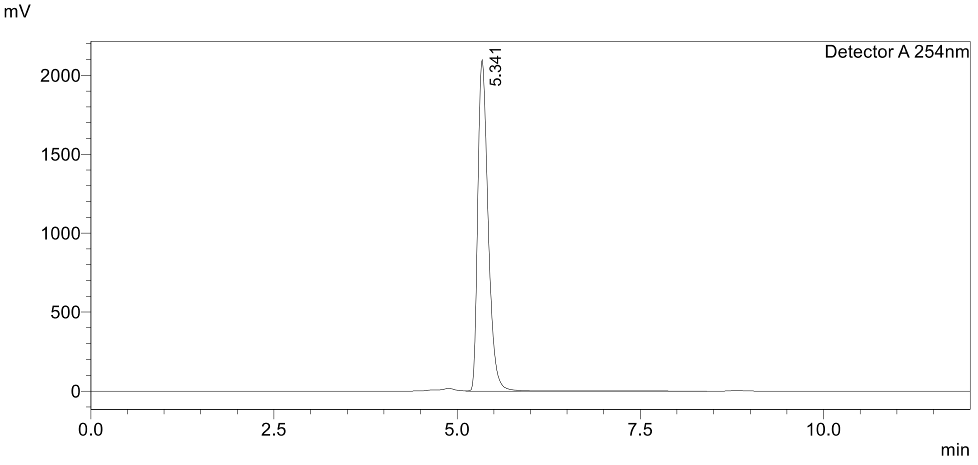

<Peak Table>

Detector A 254nm

Peak#	Ret. Time	Area	Height	Area%
1	5.341	20742923	2097279	100.000
Total		20742923	2097279	100.000

C:\LabSolutions\Data\Project1\YM-OL031-3.lcd

Analysis Report

Compound **9d**

<Sample Information>

Sample Name	: YM-OL030-4		
Sample ID	: YM-OL030-80ACN20H20-1		
Data Filename	: YM-OL030-4.lcd		
Method Filename	: method 0.5mlmin 251018.lcm		
Batch Filename	: YM-OL030-80ACN20H2O-1.lcb		
Vial #	: 1-28	Sample Type	: Unknown
Injection Volume	: 10 uL		
Date Acquired	: 17/01/2019 15:20:52	Acquired by	: System Administrator
Date Processed	: 17/01/2019 15:32:53	Processed by	: System Administrator

<Chromatogram>

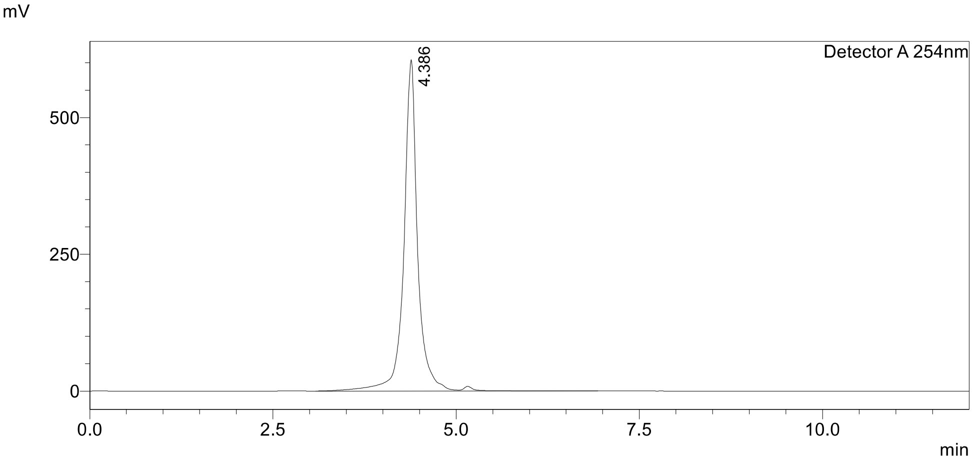

<Peak Table>

Detector A 254nm

Peak#	Ret. Time	Area	Height	Area%
1	4.386	7918973	605360	100.000
Total		7918973	605360	100.000

C:\LabSolutions\Data\Project1\YM-OL030-4.lcd

Analysis Report

<Sample Information>

Compound **9e**

Sample Name : YM-OL033-2
Sample ID : YM-OL033-80MeOH20H20-2
Data Filename : YM-OL033-2.lcd
Method Filename : method 0.5mlmin 251018.lcm
Batch Filename : YM-OL033-80MeOH20H2O-2.lcb
Vial # : 1-28
Injection Volume : 10 uL
Date Acquired : 07/01/2019 14:07:46
Date Processed : 07/01/2019 14:19:47

Sample Type : Unknown
Acquired by : System Administrator
Processed by : System Administrator

<Chromatogram>

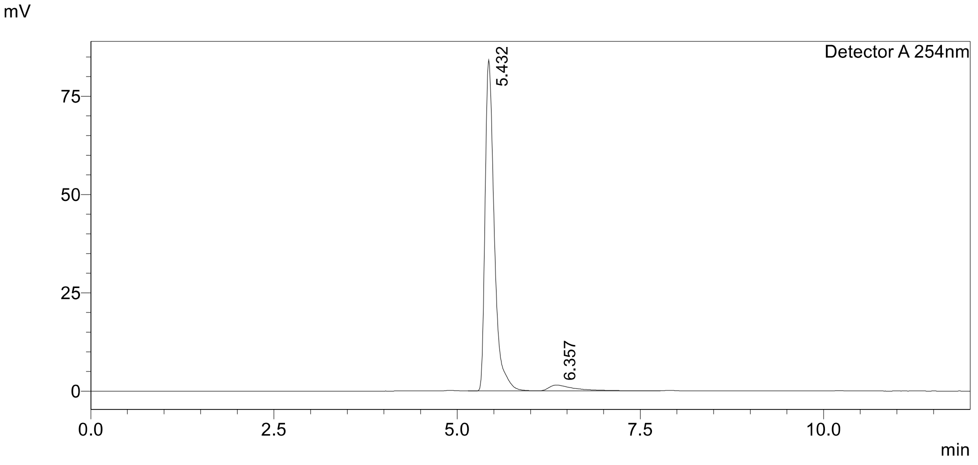

<Peak Table>

Detector A 254nm

Peak#	Ret. Time	Area	Height	Area%
1	5.432	731602	84221	94.984
2	6.357	38638	1506	5.016
Total		770239	85726	100.000

C:\LabSolutions\Data\Project1\YM-OL033-2.lcd

Analysis Report

<Sample Information>

Compound **9f**

Sample Name : YM-OL032-9
Sample ID : YM-OL032-80ACN20H20-3
Data Filename : YM-OL032-9.lcd
Method Filename : method 0.5mlmin 251018.lcm
Batch Filename : YM-OL032-80ACN20H2O-3.lcb
Vial # : 1-28
Injection Volume : 10 uL
Date Acquired : 17/01/2019 15:04:58
Date Processed : 17/01/2019 15:16:59

Sample Type : Unknown
Acquired by : System Administrator
Processed by : System Administrator

<Chromatogram>

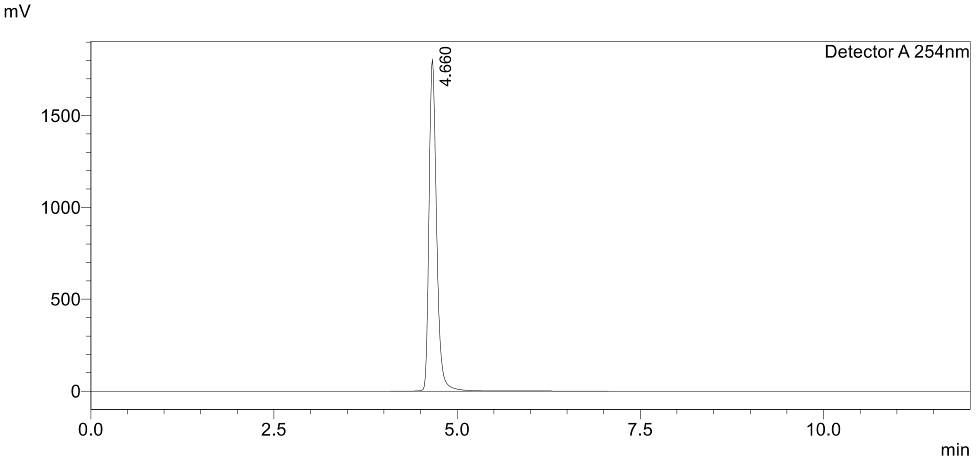

<Peak Table>

Detector A 254nm

Peak#	Ret. Time	Area	Height	Area%
1	4.660	12902828	1803357	100.000
Total		12902828	1803357	100.000
